# Comparative Risk of Alzheimer Disease and Related Dementia Among Medicare Beneficiaries With Rheumatoid Arthritis Treated With Targeted Disease-Modifying Antirheumatic Agents

**DOI:** 10.1001/jamanetworkopen.2022.6567

**Published:** 2022-04-08

**Authors:** Rishi J. Desai, Vijay R. Varma, Tobias Gerhard, Jodi Segal, Mufaddal Mahesri, Kristyn Chin, Daniel B. Horton, Seoyoung C. Kim, Sebastian Schneeweiss, Madhav Thambisetty

**Affiliations:** 1 Division of Pharmacoepidemiology and Pharmacoeconomics, Department of Medicine, Brigham and Women’s Hospital and Harvard Medical School, Boston, Massachusetts; 2 Clinical and Translational Neuroscience Section, Laboratory of Behavioral Neuroscience, National Institute on Aging, Baltimore, Maryland; 3 Center for Pharmacoepidemiology and Treatment Science, Ernest Mario School of Pharmacy, Rutgers University, New Brunswick, New Jersey; 4 Department of Medicine, Johns Hopkins University School of Medicine, Baltimore, Maryland; 5 Division of Rheumatology, Inflammation, and Immunity, Department of Medicine, Brigham and Women’s Hospital and Harvard Medical School, Boston, Massachusetts

## Abstract

**Importance:**

Cytokine signaling, including tumor necrosis factor (TNF) and interleukin (IL)-6, through the Janus-kinase (JAK)–signal transducer and activator of transcription pathway, was hypothesized to attenuate the risk of Alzheimer disease and related dementia (ADRD) in the Drug Repurposing for Effective Alzheimer Medicines (DREAM) initiative based on multiomics phenotyping.

**Objective:**

To evaluate the association between treatment with tofacitinib, tocilizumab, or TNF inhibitors compared with abatacept and risk of incident ADRD.

**Design, Setting, and Participants:**

This cohort study was conducted among US Medicare fee-for-service patients with rheumatoid arthritis aged 65 years and older from 2007 to 2017. Patients were categorized into 3 cohorts based on initiation of tofacitinib (a JAK inhibitor), tocilizumab (an IL-6 inhibitor), or TNF inhibitors compared with a common comparator abatacept (a T-cell activation inhibitor). Analyses were conducted from August 2020 to August 2021.

**Main Outcomes and Measures:**

The main outcome was onset of ADRD based on diagnosis codes evaluated in 4 alternative analysis schemes: (1) an as-treated follow-up approach, (2) an as-started follow-up approach incorporating a 6-month induction period, (3) incorporating a 6-month symptom to diagnosis period to account for misclassification of ADRD onset, and (4) identifying ADRD through symptomatic prescriptions and diagnosis codes. Hazard ratios (HRs) with 95% CIs were calculated from Cox proportional hazard regression after adjustment for 79 preexposure characteristics through propensity score matching.

**Results:**

After 1:1 propensity score matching to patients using abatacept, a total of 22 569 propensity score–matched patient pairs, including 4224 tofacitinib pairs (mean [SD] age 72.19 [5.65] years; 6945 [82.2%] women), 6369 tocilizumab pairs (mean [SD] age 72.01 [5.46] years; 10 105 [79.4%] women), and 11 976 TNF inhibitor pairs (mean [SD] age 72.67 [5.91] years; 19 710 [82.3%] women), were assessed. Incidence rates of ADRD varied from 2 to 18 per 1000 person-years across analyses schemes. There were no statistically significant associations of ADRD with tofacitinib (analysis 1: HR, 0.90 [95% CI, 0.55-1.51]; analysis 2: HR, 0.78 [95% CI, 0.53-1.13]; analysis 3: HR, 1.29 [95% CI, 0.72-2.33]; analysis 4: HR, 0.50 [95% CI, 0.21-1.20]), tocilizumab (analysis 1: HR, 0.82 [95% CI, 0.55-1.21]; analysis 2: HR, 1.05 [95% CI, 0.81-1.35]; analysis 3: HR, 1.21 [95% CI, 0.75-1.96]; analysis 4: HR, 0.78 [95% CI, 0.44-1.39]), or TNF inhibitors (analysis 1: HR, 0.93 [95% CI, 0.72-1.20]; analysis 2: HR, 1.02 [95% CI, 0.86-1.20]; analysis 3: HR, 1.13 [95% CI, 0.86-1.48]; analysis 4: 0.90 [95% CI, 0.60-1.37]) compared with abatacept. Results from prespecified subgroup analysis by age, sex, and baseline cardiovascular disease were consistent except in patients with cardiovascular disease, for whom there was a potentially lower risk of ADRD with TNF inhibitors vs abatacept, but only in analyses 2 and 4 (analysis 1: HR, 0.76 [95% CI, 0.50-1.16]; analysis 2: HR, 0.74 [95% CI, 0.56-0.99]; analysis 3: HR, 1.03 [95% CI, 0.65-1.61]; analysis 4: HR, 0.45 [95% CI, 0.21-0.98]).

**Conclusions and Relevance:**

This cohort study did not find any association of risk of ADRD in patients treated with tofacitinib, tocilizumab, or TNF inhibitors compared with abatacept.

## Key Points

**Question:**

Is use of targeted disease-modifying antirheumatic drugs associated with risk of Alzheimer disease and related dementia (ADRD)?

**Findings:**

In this cohort study including 22 569 propensity score–matched patient pairs, initiation of inhibitors of Janus-kinase, interleukin-6, or tumor necrosis factor was not associated with reduced risk of ADRD compared with initiation of abatacept, a T-cell activation inhibitor.

**Meaning:**

These results do not support advancing targeted disease-modifying antirheumatic drugs as disease modifying candidates for ADRD.

## Teaser

This cohort study compares incidence of Alzheimer disease and related dementia among Medicare patients with rheumatoid arthritis using disease-modifying antirheumatic drugs.

## Introduction

Alzheimer disease and related dementias (ADRD) remain a looming public health challenge, with current prevalence of more than 5 million in the US and with extremely limited disease-modifying treatment options.^1,2,3^ The traditional drug discovery approach of identifying targets based on experimental animal models that recapitulate the pathological features of ADRD, including amyloid plaque and neurofibrillary tangle pathologies, has had limited success.^4,5,6^ As a result, researchers increasingly recognize a need to identify early molecular triggers preceding accumulation of pathogenesis and clinical symptoms to aid in drug discovery for ADRD.^7,8^

We proposed an alternative approach to drug discovery in ADRD in the Drug Repurposing for Effective Alzheimer Medicines (DREAM) initiative,^9^ which is an ongoing multidisciplinary collaborative study aimed at identifying drug repurposing candidates for ADRD. Briefly, we generate testable hypotheses based on multiomics phenotyping of AD to identify genetic regulators of abnormal metabolic pathways associated with ADRD neuropathogenesis.^10,11^ Next, we identify existing medications with US Food and Drug Administration approval that act on the identified targets and pursue them as repurposing candidates. Finally, in a hypothesis refinement step, we conduct rigorous patient-level pharmacoepidemiologic analyses using routinely collected health care data to evaluate the association between exposure to the repurposing candidates and incident ADRD, avoiding common pitfalls of noninterventional studies, including immortal time bias and confounding by indication. Our results may lead to novel hypotheses about biological pathways and drug targets associated with ADRD that merit testing in relevant experimental models.

We recently defined a hypothetical network of interacting and intersecting metabolic pathways in AD, linked to dysregulation in brain glycolysis, the Alzheimer Disease Aberrant Metabolism (ADAM) network.^9^ We nominated genetic regulators of metabolic and signaling reactions in the ADAM network as plausible AD drug targets. In this network, cytokine signaling, including tumor necrosis factor (TNF) and interleukin (IL)-6, through the Janus-kinase–signal transducer and activator of transcription (JAK/STAT) pathway was nominated as 1 such drug target for pharmacoepidemiologic analyses in the DREAM study.

In this study, we describe results comparing the risk of ADRD in Medicare beneficiaries with rheumatoid arthritis (RA) who were treated with any of 3 targeted synthetic or biologic disease-modifying antirheumatic drugs (TDMARDs) identified in the hypothesis generation step as repurposing candidates: tofacitinib (a JAK inhibitor), tocilizumab (an IL-6 inhibitor), and TNF inhibitors, compared with an active comparator abatacept (a T-cell activation inhibitor) that is used for similar indication but was not found to be associated with changes in the JAK/STAT pathway in our omics studies. We prespecified all our hypotheses and design choices in our prior publication^9^ to safeguard against publication bias and data dredging.^12,13^

## Methods

This cohort study was approved by the Brigham and Women’s Hospital institutional review board. All the analyses were conducted using anonymized patient data, therefore the institutional review board waived the requirement for informed consent. The full study protocols for patient-level analyses in Medicare claims were preregistered on ClinicalTrials.gov prior to data analysis (ClinicalTrials.gov Identifiers: NCT04529902, NCT04529876, and NCT04529863) and contain detailed information on implementation, including all codes that were used to identify study variables to allow for independent replication and validation. This study is reported following the Strengthening the Reporting of Observational Studies in Epidemiology (STROBE) reporting guideline.

### Data Source

We used Medicare fee-for-service claims data from 2007 through 2017. Medicare Part A (hospitalizations), B (medical services), and D (prescription medications) claims are available for research purposes through the Centers for Medicare & Medicaid Services (CMS).

### Study Cohort

A new user, active comparator, observational cohort study design was used in which patients were required to have 365 days of continuous enrollment in Medicare parts A, B, and D before initiation date of study medications of interest. Date of initiation of the TDMARD was defined as the cohort entry date, and the 365-day period prior to cohort entry was defined as the baseline period. Based on the TDMARD initiated, patients were identified in 3 cohorts: tofacitinib vs abatacept, tocilizumab vs abatacept, and TNF inhibitors vs abatacept. We restricted entry to patients with at least 1 diagnosis code of RA during the baseline period and required no prior use of the study TDMARDs in each comparison. To focus on incident events, we excluded patients with existing diagnoses of ADRD any time prior to and including cohort entry date. Patients with a nursing home admission 365 days prior to and including the cohort entry date were excluded, as medication records for short nursing home stays are unavailable in Medicare claims. eFigure 1 in the Supplement shows the study design.

### Outcome Measurement

The outcome of interest was time to incident ADRD and was operationalized based on diagnosis codes recorded on inpatient or outpatient claims of AD, vascular dementia, senile, presenile, or unspecified dementia, or dementia in other diseases classified elsewhere (eTable 1 in the Supplement). Medicare claims–based dementia identification is reported to have a positive predictive value in the range of 65% to 78% when validated against a structured in-home dementia assessment.^14^ We evaluated time to incident AD as a secondary outcome of interest.

### Alternative Analytic Approaches

Pharmacoepidemiologic investigations focused on ADRD risk face numerous uncertainties, including informative censoring, reverse causality bias, and outcome misclassification. To address these concerns, we used the following 4 alternative analyses with equal priority (eFigure 2 in the Supplement).

#### Analysis 1: As-Treated Follow-up Approach

In analysis 1, the follow-up started on the day following the cohort entry date and continued until first of the following events: outcome, treatment discontinuation or switch to the comparator treatment, insurance disenrollment, death, or administrative end point (December 2017). A 90-day grace period after the end of the expected days’ supply of the most recently filled prescription was used to define the treatment discontinuation date to accommodate suboptimal adherence during treatment periods.

#### Analysis 2: As-Started Follow-up Approach Incorporating a 6-Month Induction Period

In analysis 2, we incorporated a 6-month induction period after the cohort entry date before beginning the follow-up for ADRD and followed patients for a maximum of 3 years regardless of subsequent treatment changes or discontinuation, similar to an intent-to-treat approach in randomized clinical trials. This follow-up approach addresses concerns related to informative censoring, which occurs if patients discontinue or if physicians deprescribe the treatments under consideration because of memory problems associated with ADRD but there is no claim for this diagnosis until after the treatment is discontinued. By disregarding early events, the 6-month induction period addresses reverse causation bias concerns if treatment decisions were driven by early disease symptoms, but disease diagnosis is only recorded shortly after treatment initiation.

#### Analysis 3: Incorporating a 6-Month Symptoms to Diagnosis Period

In analysis 3, we assigned an outcome date that was 6 months before the first recorded ADRD date and excluded last 6 months of follow-up for those who were censored without an event to account for the possibility that ADRD symptoms appeared some time before a diagnosis was indicated in insurance records, which could lead to misclassification of ADRD onset, and followed patients in an as-treated follow-up scheme.

#### Analysis 4: Alternate Outcome Definition

In analysis 4, the outcome was defined using a combination of diagnosis code and at least 1 prescription claim for a symptomatic treatment (ie, donepezil, galantamine, rivastigmine, and memantine) occurring within 6 months of each other, with the outcome date assigned to the second event in the sequence, and patients were observed in an as-treated follow-up scheme. Use of medication records to identify dementia had a positive predictive value exceeding 95% in a previous validation study.^15^

### Covariates

All covariates were measured in the 365-day baseline period preceding cohort entry. They included: sociodemographic factors, including age, sex, race, and receipt of low-income subsidy; risk factors for ADRD, including diabetes, stroke, and depression^16,17,18^; lifestyle factors, such as smoking, as well as use of preventive services, like screening mammography and vaccinations, to account for healthy-user confounding^19^; measures of health care services use before cohort entry, including number of prescriptions filled, number of emergency department visits, hospitalizations, and physician office visits, to minimize the potential of differential surveillance bias,^20^ and a frailty indicator^21^; RA-related treatments, including number of nonbiologic DMARDs, number of TDMARDs, and steroid and opioid use to account for differences in RA activity at baseline; and other comorbid conditions and comedications (eTable 2 and eTable 3 in the Supplement). Race data from Medicare claims are based on voluntary report from enrollees to the Social Security Administration. These data are collected by CMS for administrative reasons and released for research purposes.

### Statistical Analysis

We used propensity score (PS)^22^ matching to account for measured confounding in this study. The PSs were calculated as the projected probability of initiating the exposure of interest vs the reference drug conditional on baseline covariates using multivariable logistic regression separately for each comparison. For all of our analyses, initiators of each exposure of interest were matched with initiators of abatacept based on their PS using a nearest-neighbor algorithm within a caliper of 0.025 on the natural scale of the PS.^23,24^ Multiple diagnostics for PS analysis were evaluated including PS distributional overlap before and after matching to ensure comparability of these groups^25^ and balance in each individual covariate between 2 treatment groups using standardized differences.^26^

In the PS-matched sample, incidence rates, along with 95% CIs, for the outcome were estimated for the treatment and reference groups. We calculated cumulative incidence using cumulative incidence functions that account for competing risk by death and provided cause-specific hazard ratios (HRs) from Cox proportional hazards regression models.^27^ Prespecified subgroup analyses were conducted based on age, sex, and baseline cardiovascular disease, as there is evidence of potentially heterogenous pathogenesis of ADRD based on these factors.^28,29,30^ Statistical significance was determined as 95% CIs that did not cross 1. Statistical analyses were performed in the Aetion Evidence Platform version 4.30 (Aetion), including R version 3.4.2 (R Project for Statistical Computing), which has been scientifically validated by accurately repeating a range of previously published studies^31^ and by replicating^32^ or projecting clinical trial findings.^33^

## Results

### Cohort Characteristics

After 1:1 PS matching to patients using abatacept, a total of 22 569 PS-matched patient pairs, including 4224 tofacitinib pairs (mean [SD] age 72.19 [5.65] years; 6945 [82.2%] women), 6369 tocilizumab pairs (mean [SD] age 72.01 [5.46] years; 10 105 [79.4%] women), and 11 976 TNF inhibitor pairs (mean [SD] age 72.67 [5.91] years; 19 710 [82.3%] women), were assessed (Table 1; eFigure 3, eTable 2 and eTable 3 in the Supplement). Diabetes and hypertension were commonly observed in all 3 study cohorts. Importantly, nearly three-fourths of patients had no use of any previous TDMARDs (ie, TDMARD naive) in the TNF inhibitor cohort; while only 34% of patients in the tofacitinib cohort and 27% of patients in the tocilizumab cohort were TDMARD naive, indicating that these 2 drugs are frequently used as second-line medications. All characteristics were well-balanced after PS matching, with standardized differences <0.1 (Table 1).

**Table 1.** Select Baseline Characteristics of Patients Included in the Study Cohort After 1:1 Propensity Score Matching, Medicare Data 2007-2017

Characteristic	Patients, No (%)								
	Tofacitinib (n = 4224)	Abatacept (n = 4224)	Standardized difference	Tocilizumab (n = 6369)	Abatacept (n = 6369)	Standardized difference	TNF inhibitors (n = 11 976)	Abatacept (n = 11 976)	Standardized difference
Age, mean (SD), y	72.18 (5.70)	72.19 (5.59)	0	72.05 (5.53)	71.99 (5.38)	0.01	72.68 (5.90)	72.67 (5.92)	0
Sex									
Women	3473 (82.2)	3472 (82.2)	0	5036 (79.1)	5069 (79.6)	−0.01	9870 (82.4)	9840 (82.2)	0.01
Men	751 (17.8)	752 (17.8)	0	1333 (20.9)	1300 (20.4)	0.01	2106 (17.6)	2136 (17.8)	−0.01
Race									
American Indian	27 (0.6)	28 (0.7)	−0.01	32 (0.5)	35 (0.5)	0	38 (0.3)	44 (0.4)	−0.02
Asian	92 (2.2)	97 (2.3)	−0.01	64 (1.0)	65 (1.0)	0	142 (1.2)	154 (1.3)	−0.01
Black	442 (10.5)	434 (10.3)	0.01	371 (5.8)	365 (5.7)	0	813 (6.8)	791 (6.6)	0.01
Hispanic	132 (3.1)	137 (3.2)	−0.01	148 (2.3)	148 (2.3)	0	349 (2.9)	344 (2.9)	0
White	3408 (80.7)	3401 (80.5)	0.01	5624 (88.3)	5627 (88.3)	0	10 413 (86.9)	10 414 (87.0)	0
Unknown	41 (1.0)	37 (0.9)	0.01	41 (0.6)	39 (0.6)	0	45 (0.4)	55 (0.5)	−0.01
Other^a^	82 (1.9)	90 (2.1)	−0.01	89 (1.4)	90 (1.4)	0	176 (1.5)	174 (1.5)	0
Low income subsidy	1396 (33.0)	1383 (32.7)	0.01	966 (15.2)	974 (15.3)	0	2080 (17.4)	2103 (17.6)	−0.01
Dementia risk factors									
Diabetes	1338 (31.7)	1329 (31.5)	0	1886 (29.6)	1859 (29.2)	0.01	3884 (32.4)	3857 (32.2)	0
Obesity	808 (19.1)	812 (19.2)	0	1102 (17.3)	1084 (17.0)	0.01	1720 (14.4)	1685 (14.1)	0.01
Hypertension	3269 (77.4)	3276 (77.6)	0	4843 (76.0)	4833 (75.9)	0	9357 (78.1)	9358 (78.1)	0
CAD	1111 (26.3)	1138 (26.9)	−0.01	1680 (26.4)	1632 (25.6)	0.02	3447 (28.8)	3479 (29.0)	0
Depression	876 (20.7)	855 (20.2)	0.01	1256 (19.7)	1286 (20.2)	−0.01	2336 (19.5)	2306 (19.3)	0.01
Anxiety	717 (17.0)	706 (16.7)	0.01	900 (14.1)	913 (14.3)	−0.01	1541 (12.9)	1556 (13.0)	0
Bipolar disorder	44 (1.0)	53 (1.3)	−0.03	72 (1.1)	75 (1.2)	−0.01	98 (0.8)	107 (0.9)	−0.01
Schizophrenia	<11^b^	12 (0.3)	−0.02	<11^b^	<11^b^	0	15 (0.1)	16 (0.1)	0
Smoker	1116 (26.4)	1130 (26.8)	−0.01	1491 (23.4)	1478 (23.2)	0	2188 (18.3)	2206 (18.4)	0
Past 1 y									
Mammography	1415 (33.5)	1390 (32.9)	0.01	2185 (34.3)	2204 (34.6)	−0.01	3875 (32.4)	3887 (32.5)	0
Colonoscopy	434 (10.3)	412 (9.8)	0.02	841 (13.2)	837 (13.1)	0	1513 (12.6)	1519 (12.7)	0
Fecal occult blood test	367 (8.7)	369 (8.7)	0	597 (9.4)	615 (9.7)	−0.01	1171 (9.8)	1177 (9.8)	0
Influenza vaccination	2791 (66.1)	2772 (65.6)	0.01	4412 (69.3)	4454 (69.9)	−0.01	8092 (67.6)	8120 (67.8)	0
RA-related factors									
Targeted DMARDs, No.									
0	1454 (34.4)	1417 (33.5)	0.02	1726 (27.1)	1686 (26.5)	0.01	8791 (73.4)	8776 (73.3)	0
1	1513 (35.8)	1514 (35.8)	0	3007 (47.2)	3027 (47.5)	−0.01	2148 (17.9)	2130 (17.8)	0
2	866 (20.5)	873 (20.7)	0	1211 (19.0)	1245 (19.5)	−0.01	749 (6.3)	779 (6.5)	−0.01
≥3	391 (9.3)	420 (9.9)	−0.02	425 (6.7)	411 (6.5)	0.01	288 (2.4)	291 (2.4)	0
Nonbiologic DMARDs, No.									
0	396 (9.4)	416 (9.8)	−0.01	642 (10.1)	622 (9.8)	0.01	1079 (9.0)	1055 (8.8)	0.01
1	1542 (36.5)	1494 (35.4)	0.02	2657 (41.7)	2678 (42.0)	−0.01	4723 (39.4)	4624 (38.6)	0.02
2	1312 (31.1)	1311 (31.0)	0	1875 (29.4)	1885 (29.6)	0	3679 (30.7)	3800 (31.7)	−0.02
≥3	974 (23.1)	1003 (23.7)	−0.01	1195 (18.8)	1184 (18.6)	0.01	2495 (20.8)	2497 (20.9)	0
Opioids	2870 (67.9)	2877 (68.1)	0	4544 (71.3)	4502 (70.7)	0.01	8799 (73.5)	8742 (73.0)	0.01
Glucocorticoids	3127 (74.0)	3099 (73.4)	0.01	4913 (77.1)	4877 (76.6)	0.01	8880 (74.1)	8943 (74.7)	−0.01
Comorbid conditions									
AF	451 (10.7)	435 (10.3)	0.01	741 (11.6)	720 (11.3)	0.01	1514 (12.6)	1528 (12.8)	−0.01
Heart failure	522 (12.4)	526 (12.5)	0	748 (11.7)	697 (10.9)	0.03	1748 (14.6)	1696 (14.2)	0.01
Stroke or TIA	343 (8.1)	343 (8.1)	0	600 (9.4)	599 (9.4)	0	1098 (9.2)	1086 (9.1)	0
PVD	542 (12.8)	537 (12.7)	0	715 (11.2)	696 (10.9)	0.01	1589 (13.3)	1578 (13.2)	0
Hyperlipidemia	2824 (66.9)	2834 (67.1)	0	4390 (68.9)	4344 (68.2)	0.02	8165 (68.2)	8199 (68.5)	−0.01
Kidney dysfunction	721 (17.1)	723 (17.1)	0	1044 (16.4)	1034 (16.2)	0.01	1972 (16.5)	1986 (16.6)	0

Abbreviations: AF, atrial fibrillation; CAD, coronary artery disease; DMARD, disease-modifying antirheumatic drug; PVD, peripheral vascular disease; RA, rheumatoid arthritis; TIA, transient ischemic attack.

^a^ The Centers for Medicare and Medicaid Services data do not provide data on who is included in the other race category.

^b^ Numbers fewer than 11 are suppressed according to data use agreement with the Centers for Medicare and Medicare Services.

### Incidence Rates of ADRD

Rates of incident ADRD ranged from a high of 14 to 18 per 1000 person-years in analysis 2 (as-started analysis) to a low of 2 to 4 per 1000 person-years in analysis 4, which used a highly specific but less sensitive outcome definition requiring ADRD treatment in addition to diagnosis codes (Table 2). Rates were generally similar within analysis schemes across the 3 treatment cohorts.

**Table 2.** Incidence Rates for Alzheimer Disease and Related Dementia Across 4 Analysis Schemes

Exposure	Patients, No.	Outcomes	Person-years, No.	Follow-up time, median (IQR), d	Incidence rate per 1000 person-years (95% CI)
**Tofacitinib vs abatacept**					
Analysis 1^a^					
Tofacitinib	4224	29	3452	171 (59-396)	8.4 (5.6-12.1)
Abatacept	4224	38	3966	199 (88-467)	9.6 (6.8-13.2)
Analysis 2^b^					
Tofacitinib	2164	47	3363	531 (230-905)	14.0 (10.3-18.6)
Abatacept	2164	63	3481	568 (244-954)	18.1 (13.9-23.2)
Analysis 3^c^					
Tofacitinib	2053	25	2014	226 (96-525)	12.4 (8.0-18.3)
Abatacept	2053	20	2065	240 (89-553)	9.7 (5.9-15)
Analysis 4^d^					
Tofacitinib	4224	<11	3469	172 (59-397)	2.0 (0.8-4.2)
Abatacept	4224	17	3986	201 (88-471)	4.3 (2.5-6.8)
**Tocilizumab vs abatacept**					
Analysis 1^a^					
Tocilizumab	6369	44	7002	216 (89-539)	6.3 (4.6-8.4)
Abatacept	6369	58	7451	235 (102-574)	7.8 (5.9-10.1)
Analysis 2^b^					
Tocilizumab	3949	121	7867	825 (393-1095)	15.4 (12.8-18.4)
Abatacept	3949	116	7874	838 (377-1095)	14.7 (12.2-17.7)
Analysis 3^c^					
Tocilizumab	3489	36	4548	301 (112-686)	7.9 (5.5-11.0)
Abatacept	3489	31	4676	304 (104-699)	6.6 (4.5-9.4)
Analysis 4					
Tocilizumab^d^	6369	20	7022	216 (89-540)	2.9 (1.7-4.4)
Abatacept	6369	28	7486	235 (103-579)	3.7 (2.5-5.4)
**TNFI vs abatacept**					
Analysis 1^a^					
TNFI	11 976	113	15 340	237 (83-603)	7.4 (6.1-8.9)
Abatacept	11 976	128	16 130	247 (103-631)	7.9 (6.6-9.4)
Analysis 2^b^					
TNFI	7940	274	17 408	1095 (485-1095)	15.7 (13.9-17.7)
Abatacept	7940	270	17 435	1095 (495-1095)	15.5 (13.7-17.5)
Analysis 3^c^					
TNFI	7117	106	11 068	329 (127-764)	9.6 (7.8-11.6)
Abatacept	7117	99	11 490	344 (115-808)	8.6 (7-10.5)
Analysis 4^d^					
TNFI	11 976	41	15 405	237 (83-606)	2.7 (1.9-3.6)
Abatacept	11 976	48	16 226	249 (103-635)	3.0 (2.2-3.9)

Abbreviation: TNFI, tumor necrosis factor inhibitor.

^a^ Analysis 1 was an as-treated follow-up approach.

^b^ Analysis 2 was an as-started follow-up approach incorporating a 6-month induction period.

^c^ Analysis 3 incorporated a 6-month symptom to diagnosis period.

^d^ Analysis 4 used an alternate outcome definition.

### Comparative Risk of ADRD

**Figure 1.**
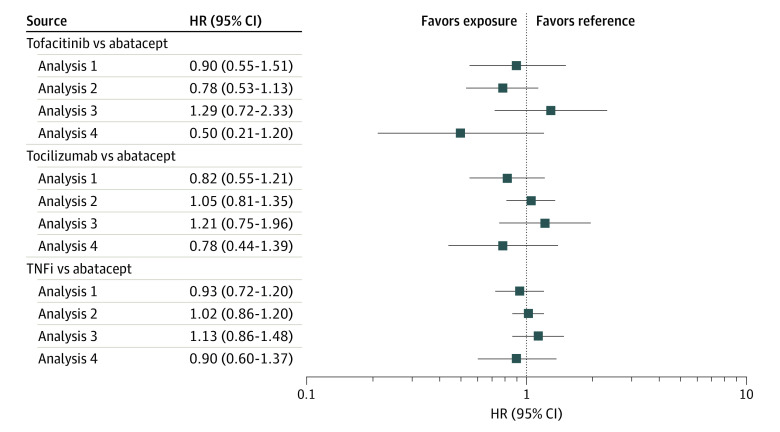
Risk of Alzheimer Disease and Related Dementia in Patients Treated With Tofacitinib, Tocilizumab, or Tumor Necrosis Factor Inhibitors (TNFi) vs Abatacept After 1:1 Propensity Score Matching, Medicare Data 2007-2017 Analysis 1 indicates an as-treated follow-up approach; analysis 2, an as-started follow-up approach incorporating a 6-month induction period; analysis 3, incorporating a 6-month symptom to diagnosis period; analysis 4, alternate outcome definition; and HR, hazard ratio.

**Figure 2.**
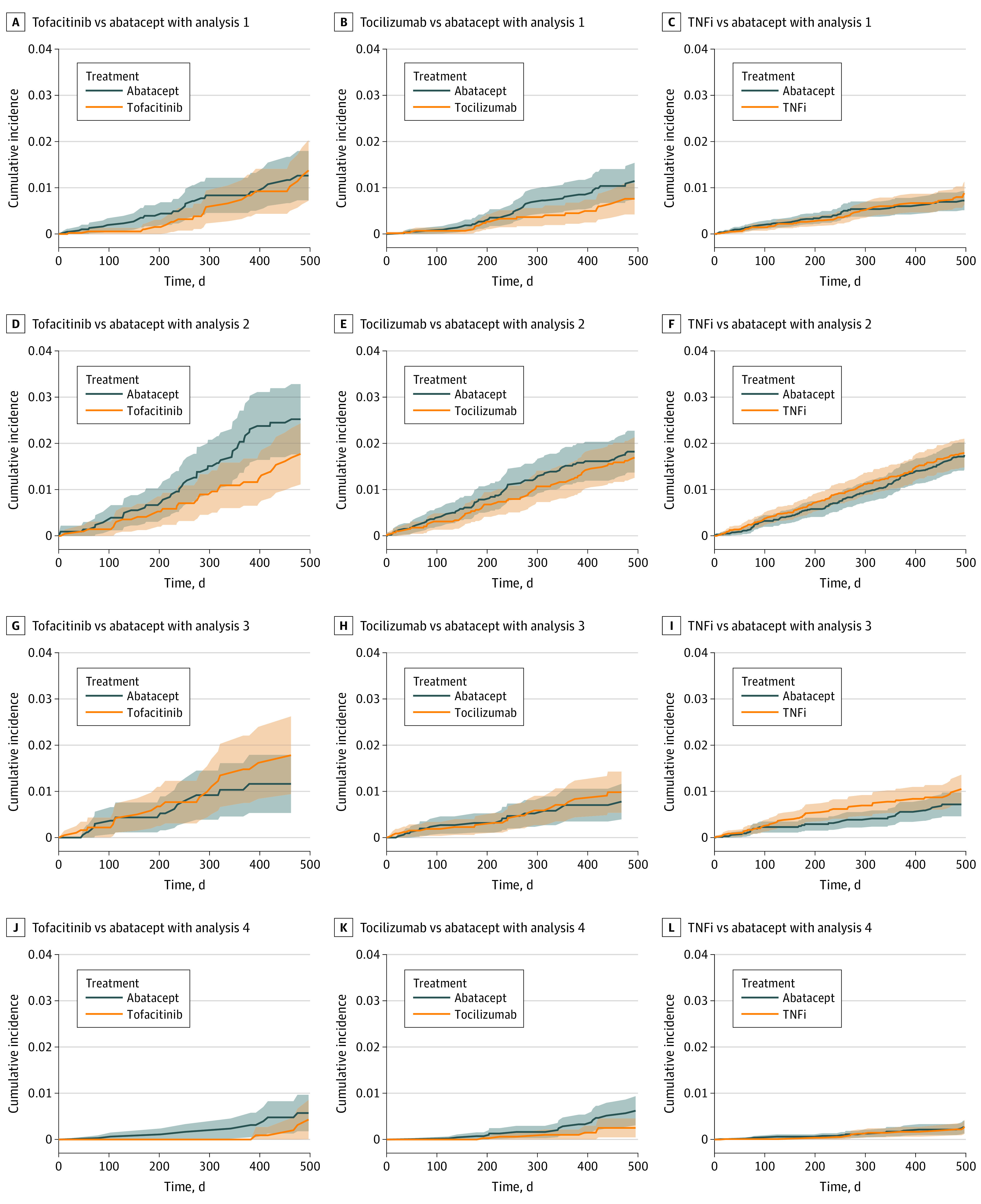
Cumulative Incidence of Alzheimer Disease and Related Dementia in Patients Treated With Tofacitinib, Tocilizumab, or Tumor Necrosis Factor Inhibitors (TNFi) vs Abatacept After 1:1 Propensity Score Matching, Medicare Data 2007-2017 Analysis 1 indicates an as-treated follow-up approach; analysis 2, an as-started follow-up approach incorporating a 6-month induction period; analysis 3, incorporating a 6-month symptom to diagnosis period; analysis 4, alternate outcome definition.

We found no evidence of differences in the risk of ADRD associated with tofacitinib (analysis 1: HR, 0.90 [95% CI, 0.55-1.51]; analysis 2: HR, 0.78 [95% CI, 0.53-1.13]; analysis 3: HR, 1.29 [95% CI, 0.72-2.33]; analysis 4: HR, 0.50 [95% CI, 0.21-1.20]), tocilizumab (analysis 1: HR, 0.82 [95% CI, 0.55-1.21]; analysis 2: HR, 1.05 [95% CI, 0.81-1.35]; analysis 3: HR, 1.21 [95% CI, 0.75-1.96]; analysis 4: HR, 0.78 [95% CI, 0.44-1.39]), or TNF inhibitors (analysis 1: HR, 0.93 [95% CI, 0.72-1.20]; analysis 2: HR, 1.02 [95% CI, 0.86-1.20]; analysis 3: HR, 1.13 [95% CI, 0.86-1.48]; analysis 4: 0.90 [95% CI, 0.60-1.37]) compared with abatacept (Figure 1; eFigure 4 in the Supplement). Estimates across all 4 analysis schemes generally were in agreement, indicating limited association of various assumptions with study results, although 95% CIs were wide for analysis 4 owing to fewer events. There were no differences in the cumulative incidence of ADRD in any of the 3 exposure groups compared with the abatacept exposed group (Figure 2). Results for the secondary outcome of AD were also consistent (eTable 4 in the Supplement).

### Subgroup Analyses

Overall, while precision was limited owing to small event counts for tofacitinib and tocilizumab cohorts, no differences were observed consistently across the 4 analysis schemes for any subgroups for these 2 comparisons (eFigure 5 and eFigure 6 in the Supplement). For the TNF inhibitor vs abatacept comparison, results from all subgroups were consistent with the primary analysis. However, for the subgroup of patients with baseline cardiovascular disease, point estimates indicated a lower incidence of ADRD with TNF inhibitors in 2 of 4 analyses (analysis 1: HR, 0.76 [95% CI, 0.50-1.16]; analysis 2: HR, 0.74 [95% CI, 0.56-0.99]; analysis 3: HR. 1.03 [95% CI, 0.65-1.61]; analysis 4: HR, 0.45 [95% CI, 0.21-0.98]) (eFigure 7 in the Supplement).

## Discussion

In this population-based cohort study designed to evaluate a prespecified hypothesis generated based on a multiomics approach, we found no reduction in the risk of ADRD for users of tofacitinib, tocilizumab, or TNF inhibitors compared with an active comparator, abatacept. These results were consistent across a range of analytic approaches, providing clear evidence that various design decisions had limited impact on the overall conclusion.

Multiple previous investigations using routine health care data have attempted to quantify the associations between TDMARDs, specifically TNF inhibitors, and reduction in ADRD risk, and have reported large effect sizes, ranging from 30% to 70% reduction compared with nonuse.^34,35^ These implausibly large effect sizes are likely attributable to combinations of various sources of bias identified in these studies, including immortal time bias, reverse causation bias, and severe confounding by indication.^9^ In this study, we attempted to address these biases using appropriate study design principles, including an active comparator and a new user design.^36,37^ We further explicitly recognized that evaluating ADRD incidence is challenging using health care claims, and there may not be a single ideal approach to address all issues. To accommodate various challenges, including potential for reverse causation bias caused by treatment selection after symptom onset but before diagnosis is made, misclassification of ADRD onset due to a time lag between symptoms and diagnosis, and limited specificity of diagnosis codes, we prespecified a series of analyses in which all these assumptions were varied to evaluate their impact on the study results. The careful attention to various sources of biases represents a key strength of our study compared with previous investigations and likely explains divergent results from previous studies. We observed results indicating potentially lower risk of ADRD with TNF inhibitors in patients with a history of cardiovascular disease in a subgroup analysis. While these results must be interpreted with caution owing to limitations associated with subgroup analyses, including possibility of type I error due to multiple hypothesis testing,^38^ further studies in diverse cohorts may clarify whether the interaction of cardiovascular risk factors and TNF signaling is associated with differentially modulating risk of ADRD.

The biological rationale underlying the potential role of TDMARDs as disease-modifying ADRD treatments is based on their ability to lower inflammation in the brain or through attenuation of systemic inflammation by specific pathways. Tofacitinib, the JAK inhibitor tested in our study, is central nervous system (CNS)–penetrant and may therefore be expected to exert direct effects on the JAK/STAT signaling pathway in the brain.^39^ On the other hand, while some of the drugs we tested, such as tocilizumab and etanercept, have poor CNS penetration, their ability to lower systemic inflammation has been proposed as a plausible mechanism to attenuate brain microglial activation, which is implicated in the pathogenesis of ADRD.^40,41^ Lending support to this hypothesis are prior positron emission tomography studies in humans and nonhuman primates with systemic inflammation that have shown evidence of increased microglial activation.^40,42^ The potential effect of etanercept on attenuating microglial activation through lowering of systemic inflammation was also the basis for a previous phase 2 trial in AD demonstrating its safety and tolerability.^43^

The interpretation of our results requires a nuanced discussion, as null findings observed in our investigation may be driven by several factors operating simultaneously. First, it is possible that TDMARDs targeting JAK, IL-6, or TNF, may truly have no causal impact on the risk or trajectory of ADRD. Second, it must be noted that we compared the risk of ADRD associated with initiation of inhibitors of these specific enzymes or cytokines with a common active comparator, abatacept, a T-cell costimulation blocker. Since abatacept has potent impact on lowering inflammation in RA, similar to the other study drugs, an alternate interpretation is that all these agents may lower ADRD risk to a similar extent. While an active comparator design complicates interpretation, we believe that it is impossible to conduct an unbiased investigation for this research question without using a truly equivalent comparator drug that is used for a similar indication and at a similar stage of the underlying illness (RA). Comparing TDMARDs with nonuse or even with nonbiologic DMARDs is subject to severe bias due to confounding, as the decision to initiate treatment with TDMARDs in old age is likely influenced by RA activity and frailty, which cannot be fully measured with claims data.^44^ Some active comparators, for instance methotrexate, which was used in another recent study as a comparator to TNF inhibitors,^45^ are likely more appropriate than nonuser comparisons owing to improved confounding adjustment. Indeed, the study comparing TNF inhibitors to methotrexate found no differences in the risk of dementia, unlike previous studies comparing TNF inhibitors with nonuse.^34,35^

### Limitations

There are certain limitations inherent to the design of this study, which could also influence interpretation. First, despite accumulating the largest cohorts to date studying this question, the number of outcomes were small for tofacitinib and tocilizumab, partly owing to short mean follow-up duration, which could mean our study was underpowered to detect smaller magnitude differences or delayed treatment outcomes. It is important to note that pathogenesis of ADRD may begin many years before a clinical diagnosis. Given the pathophysiological characteristics and prolonged preclinical phase of ADRD, longer periods of treatment and/or observation may be needed to draw firmer conclusions about the null findings. Second, although we tried addressing limitations related to identifying ADRD in health care claims through careful design, there remains a possibility of bias owing to outcome misclassification. Third, while active comparator designs using an equivalent reference exposure are less prone to confounding, these designs are not free from confounding by indication. Despite all these limitations, our results likely indicate that the large signals observed in previous studies^34,35^ may represent design artifacts rather than true associations. The current evidence base, which is substantially enhanced by our study, does not support testing TDMARDs in prospective trials despite promising biological data.^46,47^

## Conclusions

This cohort study found no differences in the risk of ADRD in patients treated with tofacitinib, tocilizumab, or TNF inhibitors compared with those treated with abatacept. Careful design choices and nuanced interpretation of results are important for generating valid, actionable evidence on drug repurposing questions explored using routine health care data.

## Notes

Supplement. **eFigure 1.** Study Design **eFigure 2.** Alternative Analytic Approaches **eFigure 3.** Patient Attrition Flowchart **eFigure 4.** Risk of Alzheimer Disease and Related Dementia in Patients Treated With Tofacitinib, Tocilizumab, or TNF Inhibitors vs Abatacept Before Propensity Score Matching **eFigure 5.** Subgroup Analyses Risk of Alzheimer Disease and Related Dementia in Patients Treated With Tofacitinib vs Abatacept After Propensity Score Matching Within Each Subgroup **eFigure 6.** Subgroup Analyses Risk of Alzheimer Disease and Related Dementia in Patients Treated With Tocilizumab vs Abatacept After Propensity Score Matching Within Each Subgroup **eFigure 7.** Subgroup Analyses Risk of Alzheimer Disease and Related Dementia in Patients Treated With TNF Inhibitors vs Abatacept After Propensity Score Matching Within Each Subgroup **eTable 1.** Codes Used to Identify Alzheimer Disease and Related Dementia From Medicare Claims **eTable 2.** Baseline Characteristics of Patients Included in the Study Cohort Before Propensity Score Matching **eTable 3.** Baseline Characteristics of Patients Included in the Study Cohort After 1:1 Propensity Score Matching **eTable 4.** Results for Alzheimer Disease in Patients Treated With Targeted Disease Modifying Antirheumatic Drugs
