# Supplementary material for: Comparative Risk of Alzheimer Disease and Related Dementia Among Medicare Beneficiaries With Rheumatoid Arthritis Treated With Targeted Disease-Modifying Antirheumatic Agents
Source: JAMA Netw Open. 2022 Apr 8;5(4):e226567. doi: 10.1001/jamanetworkopen.2022.6567 (PMC8994126; doi:10.1001/jamanetworkopen.2022.6567)
Supplement: Supplement. — eFigure 1. Study Design eFigure 2. Alternative Analytic Approaches eFigure 3. Patient Attrition Flowchart eFigure 4. Risk of Alzheimer Disease and Related Dementia in Patients Treated With Tofacitinib, Tocilizumab, or TNF Inhibitors vs Abatacept Before Propensity Score Matching eFigure 5. Subgroup Analyses Risk of Alzheimer Disease and Related Dementia in Patients Treated With Tofacitinib vs Abatacept After Propensity Score Matching Within Each Subgroup eFigure 6. Subgroup Analyses Risk of Alzheimer Disease and Related Dementia in Patients Treated With Tocilizumab vs Abatacept After Propensity Score Matching Within Each Subgroup eFigure 7. Subgroup Analyses Risk of Alzheimer Disease and Related Dementia in Patients Treated With TNF Inhibitors vs Abatacept After Propensity Score Matching Within Each Subgroup eTable 1. Codes Used to Identify Alzheimer Disease and Related Dementia From Medicare Claims eTable 2. Baseline Characteristics of Patients Included in the Study Cohort Before Propensity Score Matching eTable 3. Baseline Characteristics of Patients Included in the Study Cohort After 1:1 Propensity Score Matching eTable 4. Results for Alzheimer Disease in Patients Treated With Targeted Disease Modifying Antirheumatic Drugs [file jamanetwopen-e226567-s001.pdf]

# Supplemental Online Content

Desai RJ, Varma VR, Gerhard T, et al. Comparative risk of Alzheimer disease and related dementia among Medicare beneficiaries with rheumatoid arthritis treated with targeted disease-modifying antirheumatic agents. *JAMA Netw Open*. 2022;5(4):e226567. doi:10.1001/jamanetworkopen.2022.6567

**eFigure 1.** Study Design

**eFigure 2.** Alternative Analytic Approaches

**eFigure 3.** Patient Attrition Flowchart

**eFigure 4.** Risk of Alzheimer Disease and Related Dementia in Patients Treated With Tofacitinib, Tocilizumab, or TNF Inhibitors vs Abatacept Before Propensity Score Matching

**eFigure 5.** Subgroup Analyses Risk of Alzheimer Disease and Related Dementia in Patients Treated With Tofacitinib vs Abatacept After Propensity Score Matching Within Each Subgroup

**eFigure 6.** Subgroup Analyses Risk of Alzheimer Disease and Related Dementia in Patients Treated With Tocilizumab vs Abatacept After Propensity Score Matching Within Each Subgroup

**eFigure 7.** Subgroup Analyses Risk of Alzheimer Disease and Related Dementia in Patients Treated With TNF Inhibitors vs Abatacept After Propensity Score Matching Within Each Subgroup

**eTable 1.** Codes Used to Identify Alzheimer Disease and Related Dementia From Medicare Claims

**eTable 2.** Baseline Characteristics of Patients Included in the Study Cohort Before Propensity Score Matching

**eTable 3.** Baseline Characteristics of Patients Included in the Study Cohort After 1:1 Propensity Score Matching

**eTable 4.** Results for Alzheimer Disease in Patients Treated With Targeted Disease Modifying Antirheumatic Drugs

This supplemental material has been provided by the authors to give readers additional information about their work.

© 2022 Desai RJ et al. *JAMA Network Open.*

**eFigure 1.** Study Design

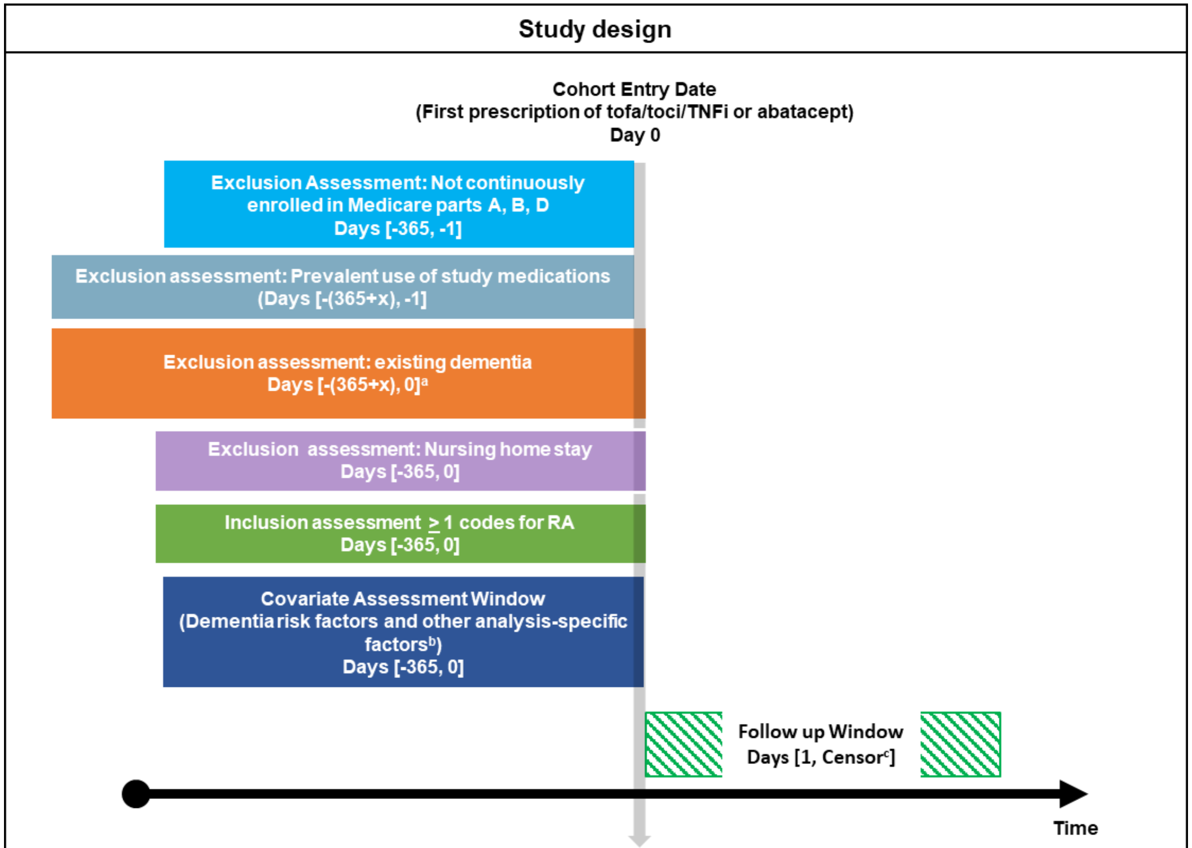


a. Existing dementia diagnosis was assessed using an 'all available lookback' approach where we excluded patients will dementia recorded any time prior to the cohort entry date
b. Covariates included dementia risk factors as well as RA-related factors, sociodemographics, and factors related to healthcare utilization
c. Earliest of: dementia onset, death, disenrollment (and switching or discontinuation of study drugs when using an 'as treated' follow-up approach, 3 years when using an intent to treat follow-up approach)

© 2022 Desai RJ et al. *JAMA Network Open.*

**eFigure 2.** Alternative Analytic Approaches

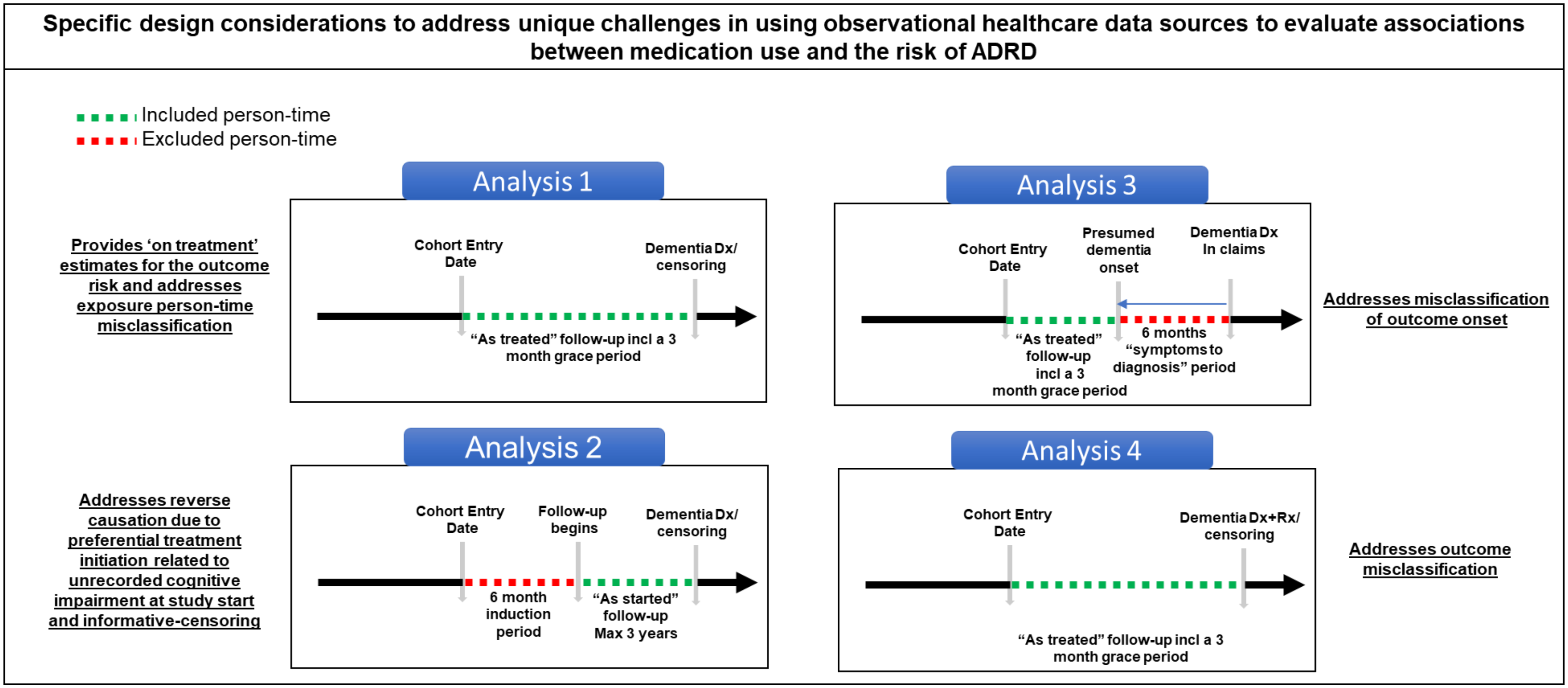


© 2022 Desai RJ et al. *JAMA Network Open.*

**eFigure 3.** Patient Attrition Flowchart

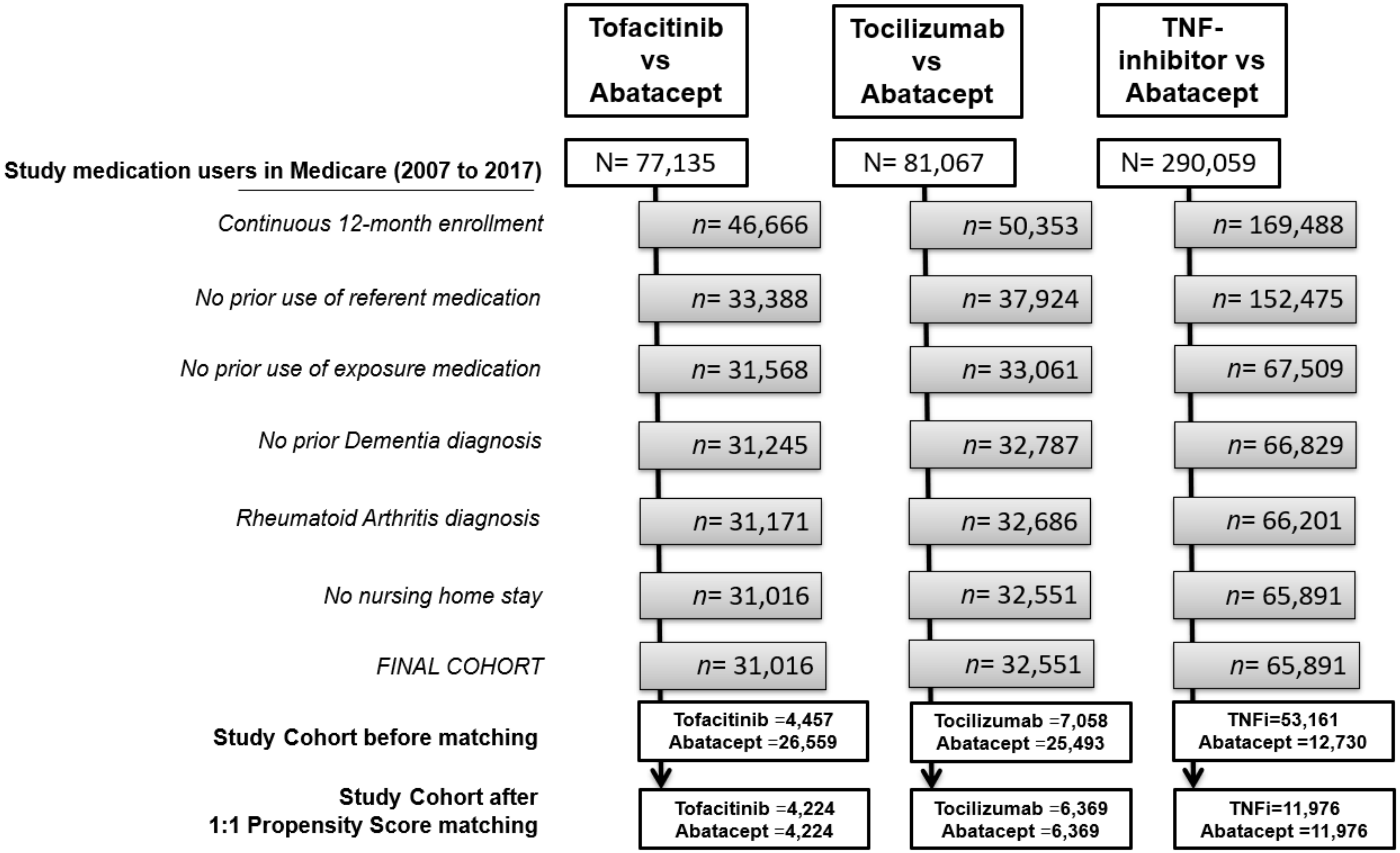


© 2022 Desai RJ et al. *JAMA Network Open.*

**eFigure 4.** Risk of Alzheimer Disease and Related Dementia in Patients Treated With Tofacitinib, Tocilizumab, or TNF Inhibitors vs Abatacept Before Propensity Score Matching

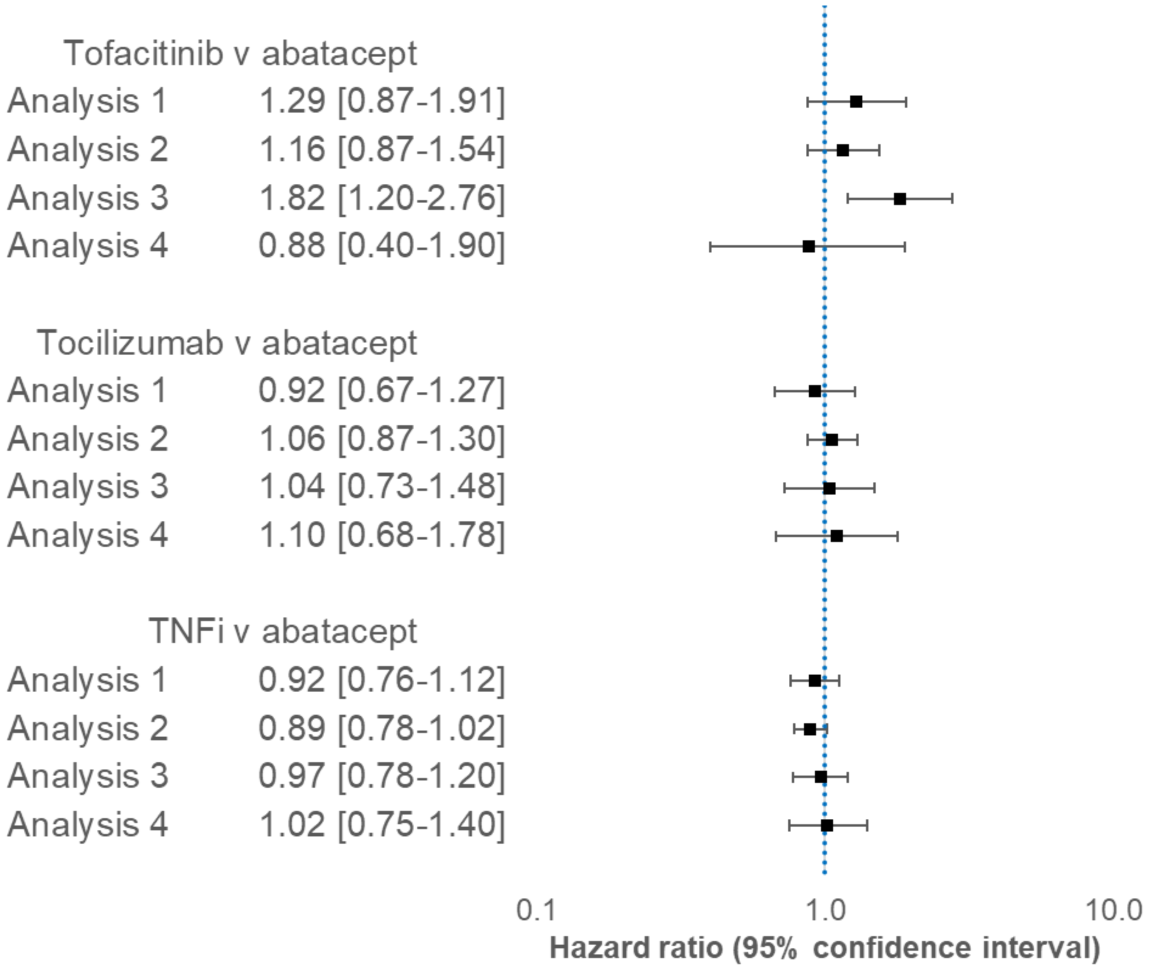


© 2022 Desai RJ et al. *JAMA Network Open.*

**eFigure 5.** Subgroup Analyses Risk of Alzheimer Disease and Related Dementia in Patients Treated With Tofacitinib vs Abatacept After Propensity Score Matching Within Each Subgroup

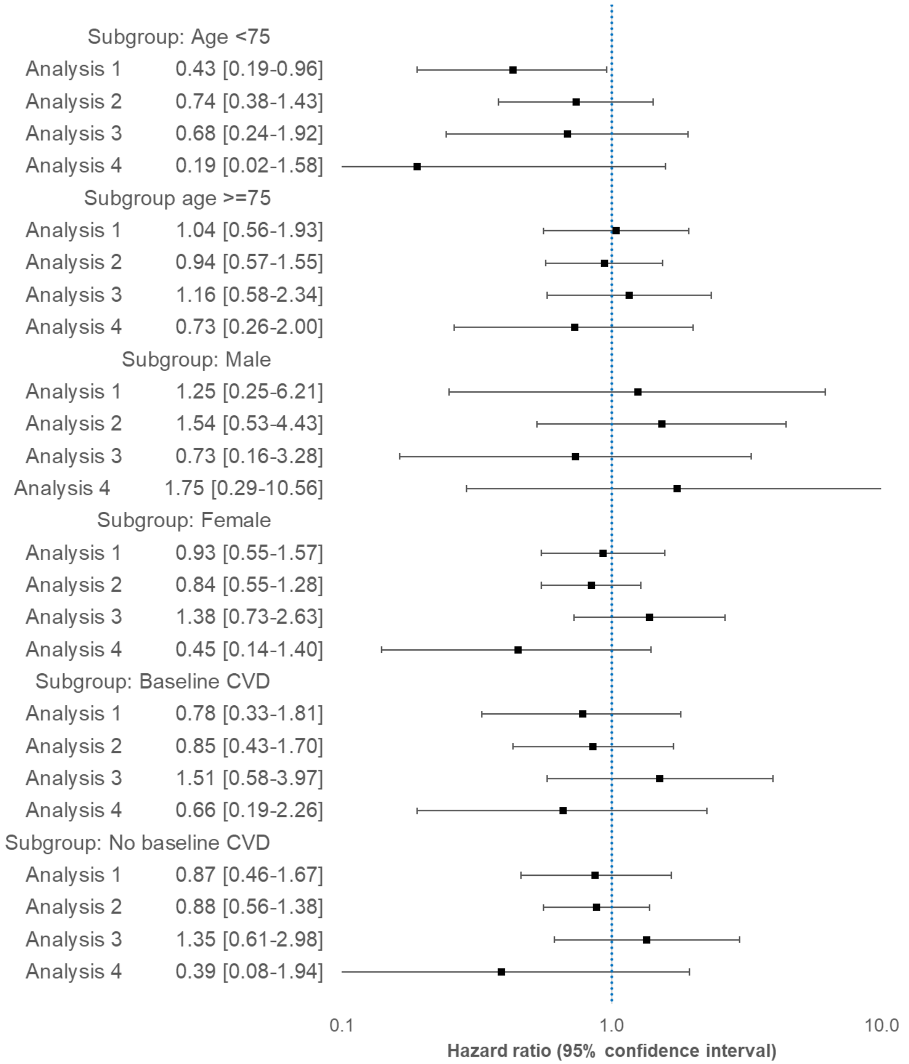


© 2022 Desai RJ et al. *JAMA Network Open.*

**eFigure 6.** Subgroup Analyses Risk of Alzheimer Disease and Related Dementia in Patients Treated With Tocilizumab vs Abatacept After Propensity Score Matching Within Each Subgroup

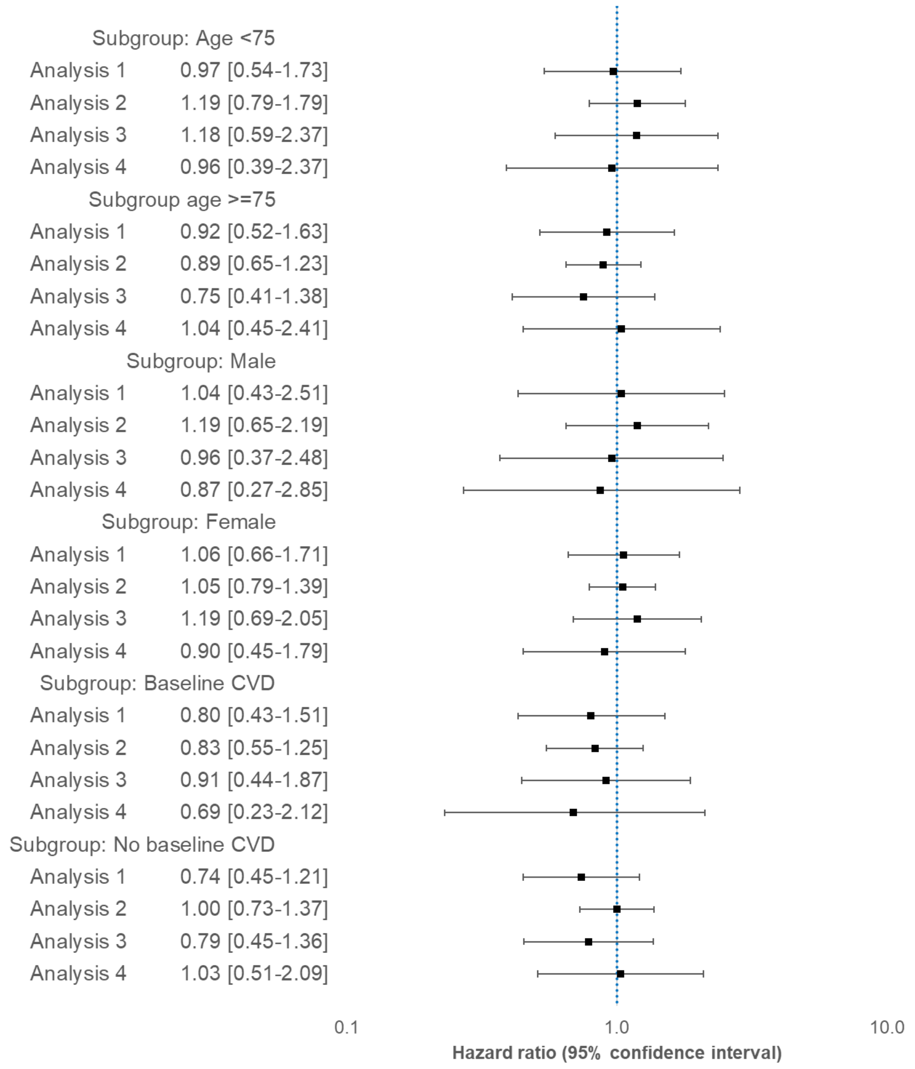


© 2022 Desai RJ et al. *JAMA Network Open.*

**eFigure 7.** Subgroup Analyses Risk of Alzheimer Disease and Related Dementia in Patients Treated With TNF Inhibitors vs Abatacept After Propensity Score Matching Within Each Subgroup

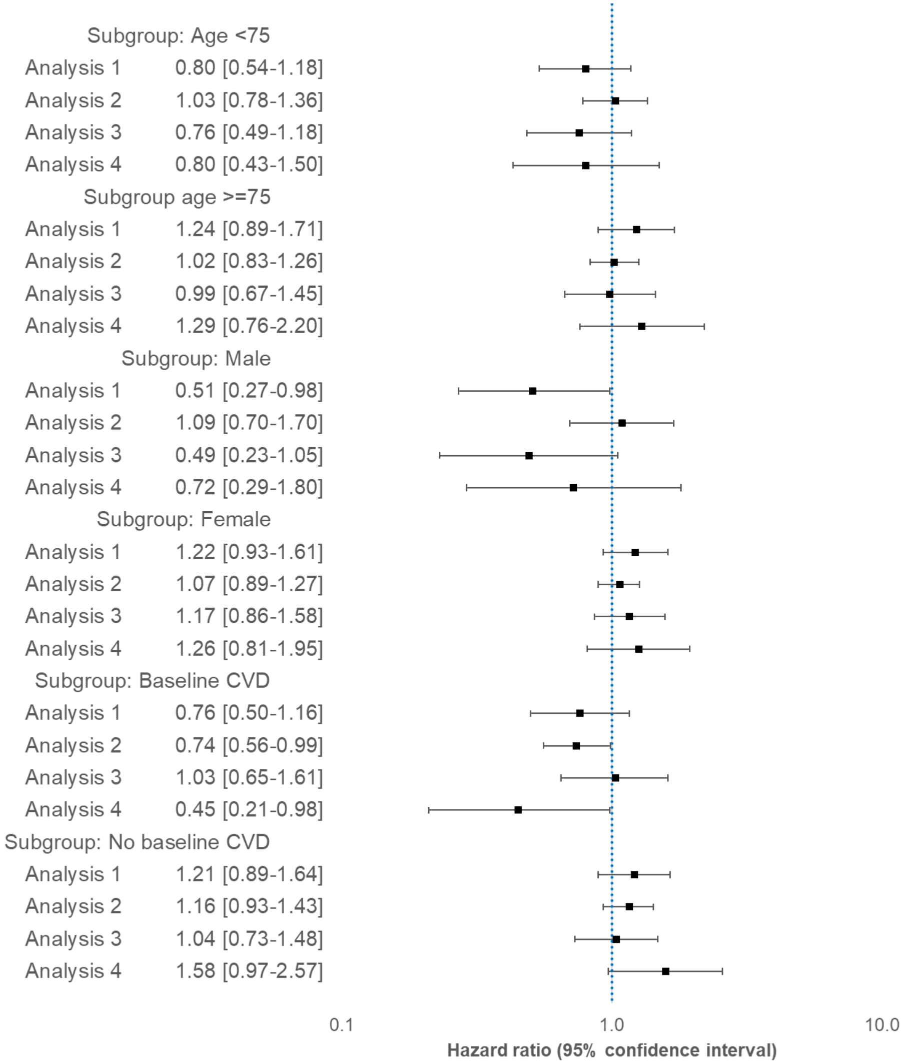


© 2022 Desai RJ et al. *JAMA Network Open.*

**eTable 1.** Codes Used to Identify Alzheimer Disease and Related Dementia From Medicare Claims

| Condition | ICD-9 and ICD-10 codes |
|---|---|
| Alzheimer's disease | 331.0*, F00*, G30* |
| Vascular dementia | 290.4*, F01* |
| Senile, presenile, or unspecified dementia | 290.0*, 290.1*. 290.3*, 797*, F03* |
| Dementia in other diseases classified elsewhere | 331.1*, 331.2*, 331.7*, 294.1*, F02* |

© 2022 Desai RJ et al. *JAMA Network Open.*

**eTable 2.** Baseline Characteristics of Patients Included in the Study Cohort Before Propensity Score Matching

| **Variable*** | **Tofacitinib** | **Abatacept** | **St. Diff** | **Tocilizumab** | **Abatacept** | **St. Diff** | **TNF inhibitors** | **Abatacept** | **St. Diff** |
|---|---|---|---|---|---|---|---|---|---|
| | **(N = 4,456)** | **(N = 25,008)** | | **(N = 6,411)** | **(N = 24,021)** | | **(N = 51,013)** | **(N = 12,000)** | |
| **Demographics** | | | | | | | | | |
| Age, mean (SD) | 72.11 (5.69) | 72.55 (5.81) | -0.08 | 72.05 (5.52) | 72.56 (5.83) | -0.09 | 71.98 (5.70) | 72.67 (5.93) | -0.12 |
| Gender, n (%) | | | | | | | | | |
| Male | 778 (17.5%) | 4,426 (17.7%) | -0.01 | 1,349 (21.0%) | 4,248 (17.7%) | 0.08 | 11,675 (22.9%) | 2,136 (17.8%) | 0.13 |
| Female | 3,678 (82.5%) | 20,582 (82.3%) | 0.01 | 5,062 (79.0%) | 19,773 (82.3%) | -0.08 | 39,338 (77.1%) | 9,864 (82.2%) | -0.13 |
| Race, n (%) | | | | | | | | | |
| White | 3,572 (80.2%) | 21,940 (87.7%) | -0.21 | 5,662 (88.3%) | 21,030 (87.5%) | 0.02 | 43,140 (84.6%) | 10,438 (87.0%) | -0.07 |
| Black | 488 (11.0%) | 1,506 (6.0%) | 0.18 | 373 (5.8%) | 1,468 (6.1%) | -0.01 | 4,012 (7.9%) | 791 (6.6%) | 0.05 |
| Asian | 96 (2.2%) | 311 (1.2%) | 0.08 | 64 (1.0%) | 307 (1.3%) | -0.03 | 893 (1.8%) | 154 (1.3%) | 0.04 |
| Hispanic | 138 (3.1%) | 675 (2.7%) | 0.02 | 148 (2.3%) | 657 (2.7%) | -0.03 | 1,493 (2.9%) | 344 (2.9%) | 0 |
| Native American | 30 (0.7%) | 116 (0.5%) | 0.03 | 32 (0.5%) | 117 (0.5%) | 0 | 401 (0.8%) | 44 (0.4%) | 0.05 |
| Other | 89 (2.0%) | 326 (1.3%) | 0.05 | 90 (1.4%) | 315 (1.3%) | 0.01 | 767 (1.5%) | 174 (1.5%) | 0 |
| Unknown | 43 (1.0%) | 134 (0.5%) | 0.06 | 42 (0.7%) | 127 (0.5%) | 0.03 | 307 (0.6%) | 55 (0.5%) | 0.01 |
| Region, n (%) | | | | | | | | | |
| Northeast | 1,029 (23.1%) | 3,665 (14.7%) | 0.22 | 1,059 (16.5%) | 3,555 (14.8%) | 0.05 | 8,838 (17.3%) | 1,785 (14.9%) | 0.07 |
| South | 1,834 (41.2%) | 11,410 (45.6%) | -0.09 | 2,761 (43.1%) | 10,938 (45.5%) | -0.05 | 21,973 (43.1%) | 5,608 (46.7%) | -0.07 |
| North Central | 720 (16.2%) | 5,277 (21.1%) | -0.13 | 1,172 (18.3%) | 5,078 (21.1%) | -0.07 | 10,303 (20.2%) | 2,452 (20.4%) | 0 |
| West | 869 (19.5%) | 4,598 (18.4%) | 0.03 | 1,418 (22.1%) | 4,392 (18.3%) | 0.09 | 9,801 (19.2%) | 2,120 (17.7%) | 0.04 |
| Unknown | <11 | 58 (0.2%) | -0.03 | <11 | 58 (0.2%) | -0.06 | 98 (0.2%) | 35 (0.3%) | -0.02 |
| Calendar year of index date, n (%) | | | | | | | | | |
| 2007 | 0 (0.0%) | 2,057 (8.2%) | -0.42 | 0 (0.0%) | 2,057 (8.6%) | -0.43 | 4,156 (8.1%) | 870 (7.2%) | 0.03 |
| 2008 | 0 (0.0%) | 1,814 (7.3%) | -0.4 | 0 (0.0%) | 1,814 (7.6%) | -0.41 | 3,294 (6.5%) | 775 (6.5%) | 0 |
| 2009 | 0 (0.0%) | 2,136 (8.5%) | -0.43 | 0 (0.0%) | 2,136 (8.9%) | -0.44 | 4,129 (8.1%) | 971 (8.1%) | 0 |
| 2010 | 0 (0.0%) | 1,959 (7.8%) | -0.41 | 62 (1.0%) | 1,958 (8.2%) | -0.35 | 4,122 (8.1%) | 948 (7.9%) | 0.01 |
| 2011 | 0 (0.0%) | 1,848 (7.4%) | -0.4 | 755 (11.8%) | 1,807 (7.5%) | 0.15 | 4,326 (8.5%) | 913 (7.6%) | 0.03 |
| 2012 | 15 (0.3%) | 2,249 (9.0%) | -0.42 | 574 (9.0%) | 2,153 (9.0%) | 0 | 4,423 (8.7%) | 1,038 (8.6%) | 0 |
| 2013 | 486 (10.9%) | 2,223 (8.9%) | 0.07 | 804 (12.5%) | 2,089 (8.7%) | 0.12 | 4,609 (9.0%) | 1,087 (9.1%) | 0 |

© 2022 Desai RJ et al. *JAMA Network Open.*

| | | | | | | | | | |
|---|---|---|---|---|---|---|---|---|---|
| 2014 | 850 (19.1%) | 2,133 (8.5%) | 0.31 | 907 (14.1%) | 1,996 (8.3%) | 0.18 | 5,003 (9.8%) | 1,026 (8.6%) | 0.04 |
| 2015 | 958 (21.5%) | 2,627 (10.5%) | 0.3 | 987 (15.4%) | 2,457 (10.2%) | 0.16 | 5,983 (11.7%) | 1,388 (11.6%) | 0 |
| 2016 | 903 (20.3%) | 2,808 (11.2%) | 0.25 | 1,043 (16.3%) | 2,610 (10.9%) | 0.16 | 5,089 (10.0%) | 1,405 (11.7%) | -0.05 |
| 2017 | 1,244 (27.9%) | 3,154 (12.6%) | 0.39 | 1,279 (20.0%) | 2,944 (12.3%) | 0.21 | 5,879 (11.5%) | 1,579 (13.2%) | -0.05 |
| Low income subsidy, n (%) | 1,600 (35.9%) | 4,409 (17.6%) | 0.42 | 972 (15.2%) | 4,360 (18.2%) | -0.08 | 12,357 (24.2%) | 2,103 (17.5%) | 0.17 |
| **Dementia risk factors, n (%)** | | | | | | | | | |
| Diabetes | 1,411 (31.7%) | 7,583 (30.3%) | 0.03 | 1,894 (29.5%) | 7,319 (30.5%) | -0.02 | 16,183 (31.7%) | 3,868 (32.2%) | -0.01 |
| Obesity | 851 (19.1%) | 3,385 (13.5%) | 0.15 | 1,115 (17.4%) | 3,180 (13.2%) | 0.12 | 6,927 (13.6%) | 1,688 (14.1%) | -0.01 |
| Hypertension | 3,452 (77.5%) | 19,114 (76.4%) | 0.03 | 4,869 (75.9%) | 18,346 (76.4%) | -0.01 | 39,242 (76.9%) | 9,380 (78.2%) | -0.03 |
| Coronary artery disease | 1,166 (26.2%) | 6,837 (27.3%) | -0.02 | 1,690 (26.4%) | 6,580 (27.4%) | -0.02 | 13,221 (25.9%) | 3,494 (29.1%) | -0.07 |
| Depression | 933 (20.9%) | 4,678 (18.7%) | 0.06 | 1,264 (19.7%) | 4,437 (18.5%) | 0.03 | 9,149 (17.9%) | 2,317 (19.3%) | -0.04 |
| Anxiety | 769 (17.3%) | 3,243 (13.0%) | 0.12 | 907 (14.1%) | 3,070 (12.8%) | 0.04 | 6,564 (12.9%) | 1,559 (13.0%) | 0 |
| Bipolar disorder | 49 (1.1%) | 241 (1.0%) | 0.01 | 73 (1.1%) | 232 (1.0%) | 0.01 | 597 (1.2%) | 108 (0.9%) | 0.03 |
| Schizophrenia | 16 (0.4%) | 33 (0.1%) | 0.06 | <11 | 31 (0.1%) | 0 | 137 (0.3%) | 16 (0.1%) | 0.04 |
| **Markers for healthy behavior, frailty, healthcare use** | | | | | | | | | |
| Smoking, n (%) | 1,187 (26.6%) | 4,485 (17.9%) | 0.21 | 1,500 (23.4%) | 4,222 (17.6%) | 0.14 | 9,287 (18.2%) | 2,213 (18.4%) | -0.01 |
| Mammography, n (%) | 1,488 (33.4%) | 8,205 (32.8%) | 0.01 | 2,197 (34.3%) | 7,835 (32.6%) | 0.04 | 15,150 (29.7%) | 3,896 (32.5%) | -0.06 |
| Colonoscopy, n (%) | 452 (10.1%) | 3,146 (12.6%) | -0.08 | 849 (13.2%) | 3,002 (12.5%) | 0.02 | 6,910 (13.5%) | 1,524 (12.7%) | 0.02 |
| Fecal occult blood test, n (%) | 383 (8.6%) | 2,401 (9.6%) | -0.03 | 604 (9.4%) | 2,313 (9.6%) | -0.01 | 4,952 (9.7%) | 1,180 (9.8%) | 0 |
| Influenza vaccination, n (%) | 2,928 (65.7%) | 17,111 (68.4%) | -0.06 | 4,438 (69.2%) | 16,425 (68.4%) | 0.02 | 33,271 (65.2%) | 8,140 (67.8%) | -0.06 |
| Pneumococcal vaccination, n (%) | 1,819 (40.8%) | 6,599 (26.4%) | 0.31 | 2,225 (34.7%) | 6,259 (26.1%) | 0.19 | 13,107 (25.7%) | 3,275 (27.3%) | -0.04 |
| Herpes zoster vaccination, n (%) | 32 (0.7%) | 122 (0.5%) | 0.03 | 22 (0.3%) | 122 (0.5%) | -0.03 | 357 (0.7%) | 77 (0.6%) | 0.01 |
| Bone mineral density test, n (%) | 1,103 (24.8%) | 7,761 (31.0%) | -0.14 | 1,863 (29.1%) | 7,473 (31.1%) | -0.04 | 14,879 (29.2%) | 3,890 (32.4%) | -0.07 |
| Number of distinct generic agents, mean (SD) | 15.99 (7.23) | 14.51 (6.69) | 0 | 14.47 (6.73) | 14.52 (6.71) | 0 | 14.23 (6.77) | 14.50 (6.63) | 0 |
| Number of emergency room visits, mean (SD) | 0.69 (1.38) | 0.65 (1.18) | 0 | 0.64 (1.21) | 0.65 (1.19) | 0 | 0.62 (1.32) | 0.67 (1.22) | 0 |
| Number of outpatient visits, mean (SD) | 18.73 (19.02) | 20.75 (17.93) | 0 | 20.27 (16.84) | 20.71 (18.06) | 0 | 19.16 (18.07) | 20.78 (17.93) | 0 |
| Number of hospitalizations, mean (SD) | 0.31 (0.84) | 0.36 (0.79) | 0 | 0.32 (0.72) | 0.36 (0.79) | 0 | 0.30 (0.71) | 0.37 (0.81) | 0 |
| Number of physician office visits, mean (SD) | 4.92 (9.57) | 5.53 (8.58) | 0 | 5.02 (8.91) | 5.57 (8.81) | 0 | 5.53 (9.70) | 5.70 (9.07) | 0 |

© 2022 Desai RJ et al. *JAMA Network Open.*

| | | | | | | | | | |
|---|---|---|---|---|---|---|---|---|---|
| Number of rheumatologist visits, mean (SD) | 4.39 (3.15) | 5.44 (4.06) | 0 | 5.53 (4.00) | 5.38 (4.03) | 0 | 4.43 (3.45) | 4.99 (3.73) | 0 |
| Number of C-reactive protein tests ordered, mean (SD) | 2.56 (2.29) | 2.79 (2.70) | 0 | 3.40 (2.99) | 2.73 (2.66) | 0 | 2.42 (2.36) | 2.66 (2.54) | 0 |
| Number of serum creatinine tests ordered, mean (SD) | 5.81 (3.38) | 6.59 (3.62) | 0 | 6.78 (3.66) | 6.52 (3.58) | 0 | 5.84 (3.38) | 6.44 (3.70) | 0 |
| Composite frailty score, mean (SD) | 0.17 (0.04) | 0.17 (0.04) | 0 | 0.17 (0.04) | 0.17 (0.04) | 0 | 0.16 (0.04) | 0.17 (0.04) | 0 |
| Osteoporosis, n (%) | 1,734 (38.9%) | 10,744 (43.0%) | -0.08 | 2,622 (40.9%) | 10,290 (42.8%) | -0.04 | 18,694 (36.6%) | 5,286 (44.0%) | -0.15 |
| Fractures, n (%) | 499 (11.2%) | 2,762 (11.0%) | 0.01 | 700 (10.9%) | 2,646 (11.0%) | 0 | 5,169 (10.1%) | 1,415 (11.8%) | -0.05 |
| Falls, n (%) | 215 (4.8%) | 1,188 (4.8%) | 0 | 305 (4.8%) | 1,135 (4.7%) | 0 | 2,239 (4.4%) | 556 (4.6%) | -0.01 |
| Use of supplemental oxygen, n (%) | 101 (2.3%) | 322 (1.3%) | 0.08 | 121 (1.9%) | 298 (1.2%) | 0.06 | 516 (1.0%) | 169 (1.4%) | -0.04 |
| Combined comorbidity score, mean (SD) | 1.59 (2.24) | 1.58 (2.23) | 0 | 1.58 (2.21) | 1.58 (2.23) | 0 | 1.40 (2.13) | 1.74 (2.32) | 0 |
| **Comedication use, n (%)** | | | | | | | | | |
| Lithium | <11 | 24 (0.1%) | 0.03 | <11 | <11 | 0 | 82 (0.2%) | <11 | 0.03 |
| Anti-epileptic mood stabilizers | 68 (1.5%) | 315 (1.3%) | 0.02 | 100 (1.6%) | 296 (1.2%) | 0.03 | 643 (1.3%) | 152 (1.3%) | 0 |
| Anti-epileptics (other than mood stabilizers) | 1,261 (28.3%) | 6,063 (24.2%) | 0.09 | 1,642 (25.6%) | 5,788 (24.1%) | 0.03 | 11,175 (21.9%) | 3,050 (25.4%) | -0.08 |
| Atypical antipsychotics | 110 (2.5%) | 368 (1.5%) | 0.07 | 105 (1.6%) | 344 (1.4%) | 0.02 | 856 (1.7%) | 186 (1.6%) | 0.01 |
| Benzodiazepines | 978 (21.9%) | 3,068 (12.3%) | 0.26 | 1,140 (17.8%) | 2,843 (11.8%) | 0.17 | 5,916 (11.6%) | 1,621 (13.5%) | -0.06 |
| Serotonin-norepinephrine reuptake inhibitors | 626 (14.0%) | 2,818 (11.3%) | 0.08 | 772 (12.0%) | 2,666 (11.1%) | 0.03 | 5,152 (10.1%) | 1,362 (11.3%) | -0.04 |
| Selective serotonin reuptake inhibitors | 879 (19.7%) | 5,133 (20.5%) | -0.02 | 1,362 (21.2%) | 4,907 (20.4%) | 0.02 | 10,074 (19.7%) | 2,502 (20.8%) | -0.03 |
| Tricyclic antidepressants | 268 (6.0%) | 1,883 (7.5%) | -0.06 | 466 (7.3%) | 1,794 (7.5%) | -0.01 | 3,410 (6.7%) | 893 (7.4%) | -0.03 |
| Typical antipsychotics | <11 | 52 (0.2%) | 0 | 12 (0.2%) | 51 (0.2%) | 0 | 111 (0.2%) | 34 (0.3%) | -0.02 |
| Anticoagulants | 472 (10.6%) | 2,718 (10.9%) | -0.01 | 721 (11.2%) | 2,596 (10.8%) | 0.01 | 4,724 (9.3%) | 1,405 (11.7%) | -0.08 |
| Antiplatelet agents | 367 (8.2%) | 2,204 (8.8%) | -0.02 | 499 (7.8%) | 2,136 (8.9%) | -0.04 | 4,303 (8.4%) | 1,114 (9.3%) | -0.03 |
| Angiotensin II receptor blockers | 1,137 (25.5%) | 5,856 (23.4%) | 0.05 | 1,533 (23.9%) | 5,603 (23.3%) | 0.01 | 11,477 (22.5%) | 2,845 (23.7%) | -0.03 |
| Angiotensin converting enzyme inhibitors | 1,182 (26.5%) | 7,120 (28.5%) | -0.04 | 1,745 (27.2%) | 6,876 (28.6%) | -0.03 | 14,943 (29.3%) | 3,443 (28.7%) | 0.01 |
| Beta blockers | 1,777 (39.9%) | 9,906 (39.6%) | 0.01 | 2,520 (39.3%) | 9,528 (39.7%) | -0.01 | 19,237 (37.7%) | 4,972 (41.4%) | -0.08 |
| Calcium channel blockers | 1,313 (29.5%) | 6,972 (27.9%) | 0.04 | 1,732 (27.0%) | 6,698 (27.9%) | -0.02 | 14,241 (27.9%) | 3,373 (28.1%) | 0 |
| Diuretics | 1,397 (31.4%) | 8,277 (33.1%) | -0.04 | 2,046 (31.9%) | 7,952 (33.1%) | -0.03 | 15,876 (31.1%) | 4,113 (34.3%) | -0.07 |
| Nitrates | 298 (6.7%) | 1,775 (7.1%) | -0.02 | 404 (6.3%) | 1,718 (7.2%) | -0.04 | 3,344 (6.6%) | 918 (7.6%) | -0.04 |
| Lipid lowering drugs | 2,245 (50.4%) | 11,919 (47.7%) | 0.05 | 3,178 (49.6%) | 11,391 (47.4%) | 0.04 | 24,485 (48.0%) | 5,877 (49.0%) | -0.02 |

© 2022 Desai RJ et al. *JAMA Network Open.*

| | | | | | | | | | |
|---|---|---|---|---|---|---|---|---|---|
| Non-insulin diabetes medications | 752 (16.9%) | 3,880 (15.5%) | 0.04 | 1,023 (16.0%) | 3,735 (15.5%) | 0.01 | 8,902 (17.5%) | 1,904 (15.9%) | 0.04 |
| Insulin | 307 (6.9%) | 1,484 (5.9%) | 0.04 | 403 (6.3%) | 1,432 (6.0%) | 0.01 | 3,371 (6.6%) | 762 (6.3%) | 0.01 |
| Antidepressants | 1,776 (39.9%) | 9,580 (38.3%) | 0.03 | 2,575 (40.2%) | 9,135 (38.0%) | 0.05 | 18,253 (35.8%) | 4,631 (38.6%) | -0.06 |
| **Rheumatoid arthritis related factors** | | | | | | | | | |
| Number of combined biologic DMARDs, n (%) | | | | | | | | | |
| 0 | 1,532 (34.4%) | 8,780 (35.1%) | -0.01 | 1,732 (27.0%) | 8,779 (36.5%) | -0.21 | 42,333 (83.0%) | 8,779 (73.2%) | 0.24 |
| 1 | 1,578 (35.4%) | 11,711 (46.8%) | -0.23 | 3,019 (47.1%) | 11,510 (47.9%) | -0.02 | 6,475 (12.7%) | 2,135 (17.8%) | -0.14 |
| 2 | 922 (20.7%) | 3,566 (14.3%) | 0.17 | 1,220 (19.0%) | 3,050 (12.7%) | 0.17 | 1,386 (2.7%) | 795 (6.6%) | -0.19 |
| 3 or more | 424 (9.5%) | 951 (3.8%) | 0.23 | 440 (6.9%) | 682 (2.8%) | 0.19 | 819 (1.6%) | 291 (2.4%) | -0.06 |
| Number of combined nonbiologic DMARDs, n (%) | | | | | | | | | |
| 0 | 420 (9.4%) | 2,363 (9.4%) | 0 | 649 (10.1%) | 2,289 (9.5%) | 0.02 | 5,392 (10.6%) | 1,056 (8.8%) | 0.06 |
| 1 | 1,613 (36.2%) | 10,332 (41.3%) | -0.1 | 2,679 (41.8%) | 9,944 (41.4%) | 0.01 | 22,286 (43.7%) | 4,627 (38.6%) | 0.1 |
| 2 | 1,398 (31.4%) | 7,573 (30.3%) | 0.02 | 1,883 (29.4%) | 7,268 (30.3%) | -0.02 | 15,346 (30.1%) | 3,809 (31.7%) | -0.03 |
| 3 or more | 1,025 (23.0%) | 4,740 (19.0%) | 0.1 | 1,200 (18.7%) | 4,520 (18.8%) | 0 | 7,989 (15.7%) | 2,508 (20.9%) | -0.13 |
| Use of other nonbiologic DMARDs, n (%) | 868 (19.5%) | 4,432 (17.7%) | 0.05 | 1,020 (15.9%) | 4,270 (17.8%) | -0.05 | 8,912 (17.5%) | 2,383 (19.9%) | -0.06 |
| Methotrexate, n (%) | 2,279 (51.1%) | 14,395 (57.6%) | -0.13 | 3,458 (53.9%) | 13,906 (57.9%) | -0.08 | 33,462 (65.6%) | 7,005 (58.4%) | 0.15 |
| Hydroxychloroquine, n (%) | 1,095 (24.6%) | 5,885 (23.5%) | 0.03 | 1,277 (19.9%) | 5,710 (23.8%) | -0.09 | 13,001 (25.5%) | 3,484 (29.0%) | -0.08 |
| Leflunomide, n (%) | 953 (21.4%) | 4,742 (19.0%) | 0.06 | 1,154 (18.0%) | 4,585 (19.1%) | -0.03 | 8,563 (16.8%) | 2,501 (20.8%) | -0.1 |
| Nonsteroidal anti-inflammatory drugs/Coxibs, n (%) | 1,578 (35.4%) | 8,423 (33.7%) | 0.04 | 2,059 (32.1%) | 8,124 (33.8%) | -0.04 | 20,621 (40.4%) | 4,123 (34.4%) | 0.12 |
| Opioids, n (%) | 3,033 (68.1%) | 18,062 (72.2%) | -0.09 | 4,574 (71.3%) | 17,320 (72.1%) | -0.02 | 35,170 (68.9%) | 8,764 (73.0%) | -0.09 |
| Glucocorticoids use/dose, n (%) | 3,292 (73.9%) | 18,512 (74.0%) | 0 | 4,945 (77.1%) | 17,784 (74.0%) | 0.07 | 36,429 (71.4%) | 8,961 (74.7%) | -0.07 |
| **Comorbid conditions, n (%)** | | | | | | | | | |
| Atrial fibrillation | 464 (10.4%) | 2,908 (11.6%) | -0.04 | 744 (11.6%) | 2,797 (11.6%) | 0 | 4,797 (9.4%) | 1,544 (12.9%) | -0.11 |
| Heart failure | 543 (12.2%) | 3,212 (12.8%) | -0.02 | 751 (11.7%) | 3,103 (12.9%) | -0.04 | 5,226 (10.2%) | 1,711 (14.3%) | -0.13 |
| Stroke or transient ischemic attack | 360 (8.1%) | 2,233 (8.9%) | -0.03 | 606 (9.5%) | 2,144 (8.9%) | 0.02 | 4,116 (8.1%) | 1,090 (9.1%) | -0.04 |
| Peripheral vascular disease | 570 (12.8%) | 3,051 (12.2%) | 0.02 | 718 (11.2%) | 2,952 (12.3%) | -0.03 | 5,913 (11.6%) | 1,586 (13.2%) | -0.05 |
| Hyperlipidemia | 2,978 (66.8%) | 16,683 (66.7%) | 0 | 4,425 (69.0%) | 15,979 (66.5%) | 0.05 | 34,197 (67.0%) | 8,217 (68.5%) | -0.03 |
| Renal dysfunction | 748 (16.8%) | 3,774 (15.1%) | 0.05 | 1,051 (16.4%) | 3,610 (15.0%) | 0.04 | 6,871 (13.5%) | 1,996 (16.6%) | -0.09 |

© 2022 Desai RJ et al. *JAMA Network Open.*

| | | | | | | | | | |
|---|---|---|---|---|---|---|---|---|---|
| Chronic liver disease | 374 (8.4%) | 2,402 (9.6%) | -0.04 | 573 (8.9%) | 2,280 (9.5%) | -0.02 | 4,605 (9.0%) | 1,247 (10.4%) | -0.05 |
| Asthma | 1,003 (22.5%) | 4,028 (16.1%) | 0.16 | 1,266 (19.7%) | 3,843 (16.0%) | 0.1 | 8,100 (15.9%) | 1,996 (16.6%) | -0.02 |
| Ischemic heart disease | 1,135 (25.5%) | 6,650 (26.6%) | -0.03 | 1,635 (25.5%) | 6,403 (26.7%) | -0.03 | 12,876 (25.2%) | 3,403 (28.4%) | -0.07 |
| Chronic obstructive pulmonary disease | 1,213 (27.2%) | 6,165 (24.7%) | 0.06 | 1,750 (27.3%) | 5,906 (24.6%) | 0.06 | 12,405 (24.3%) | 3,042 (25.4%) | -0.03 |
| Malignancy | 926 (20.8%) | 6,219 (24.9%) | -0.1 | 1,691 (26.4%) | 5,947 (24.8%) | 0.04 | 11,474 (22.5%) | 3,033 (25.3%) | -0.07 |
| Drug or alcohol abuse or dependence | 535 (12.0%) | 1,795 (7.2%) | 0.16 | 609 (9.5%) | 1,705 (7.1%) | 0.09 | 4,317 (8.5%) | 925 (7.7%) | 0.03 |
| Venous thromboembolism | 261 (5.9%) | 1,453 (5.8%) | 0 | 381 (5.9%) | 1,383 (5.8%) | 0 | 2,499 (4.9%) | 768 (6.4%) | -0.07 |

* Numbers <11 are suppressed according to Data Use Agreement with the Centers for Medicare and Medicare Services

© 2022 Desai RJ et al. *JAMA Network Open.*

**eTable 3.** Baseline Characteristics of Patients Included in the Study Cohort After 1:1 Propensity Score Matching

| Variable* | Tofacitinib | Abatacept | St. Diff | Tocilizumab | Abatacept | St. Diff | TNF inhibitors | Abatacept | St. Diff |
|---|---|---|---|---|---|---|---|---|---|
| | (N = 4,224) | (N = 4,224) | | (N = 6,369) | (N = 6,369) | | (N = 11,976) | (N = 11,976) | |
| **Demographics** | | | | | | | | | |
| Age, mean (SD) | 72.18 (5.70) | 72.19 (5.59) | 0 | 72.05 (5.53) | 71.99 (5.38) | 0.01 | 72.68 (5.90) | 72.67 (5.92) | 0 |
| Gender, n (%) | | | | | | | | | |
| Male | 751 (17.8%) | 752 (17.8%) | 0 | 1,333 (20.9%) | 1,300 (20.4%) | 0.01 | 2,106 (17.6%) | 2,136 (17.8%) | -0.01 |
| Female | 3,473 (82.2%) | 3,472 (82.2%) | 0 | 5,036 (79.1%) | 5,069 (79.6%) | -0.01 | 9,870 (82.4%) | 9,840 (82.2%) | 0.01 |
| Race, n (%) | | | | | | | | | |
| White | 3,408 (80.7%) | 3,401 (80.5%) | 0.01 | 5,624 (88.3%) | 5,627 (88.3%) | 0 | 10,413 (86.9%) | 10,414 (87.0%) | 0 |
| Black | 442 (10.5%) | 434 (10.3%) | 0.01 | 371 (5.8%) | 365 (5.7%) | 0 | 813 (6.8%) | 791 (6.6%) | 0.01 |
| Asian | 92 (2.2%) | 97 (2.3%) | -0.01 | 64 (1.0%) | 65 (1.0%) | 0 | 142 (1.2%) | 154 (1.3%) | -0.01 |
| Hispanic | 132 (3.1%) | 137 (3.2%) | -0.01 | 148 (2.3%) | 148 (2.3%) | 0 | 349 (2.9%) | 344 (2.9%) | 0 |
| Native American | 27 (0.6%) | 28 (0.7%) | -0.01 | 32 (0.5%) | 35 (0.5%) | 0 | 38 (0.3%) | 44 (0.4%) | -0.02 |
| Other | 82 (1.9%) | 90 (2.1%) | -0.01 | 89 (1.4%) | 90 (1.4%) | 0 | 176 (1.5%) | 174 (1.5%) | 0 |
| Unknown | 41 (1.0%) | 37 (0.9%) | 0.01 | 41 (0.6%) | 39 (0.6%) | 0 | 45 (0.4%) | 55 (0.5%) | -0.01 |
| Region, n (%) | | | | | | | | | |
| Northeast | 951 (22.5%) | 944 (22.3%) | 0 | 1,053 (16.5%) | 1,050 (16.5%) | 0 | 1,765 (14.7%) | 1,781 (14.9%) | -0.01 |
| South | 1,754 (41.5%) | 1,775 (42.0%) | -0.01 | 2,748 (43.1%) | 2,743 (43.1%) | 0 | 5,616 (46.9%) | 5,597 (46.7%) | 0 |
| North Central | 692 (16.4%) | 657 (15.6%) | 0.02 | 1,168 (18.3%) | 1,169 (18.4%) | 0 | 2,439 (20.4%) | 2,448 (20.4%) | 0 |
| West | 823 (19.5%) | 843 (20.0%) | -0.01 | 1,399 (22.0%) | 1,405 (22.1%) | 0 | 2,114 (17.7%) | 2,115 (17.7%) | 0 |
| Unknown | <11 | <11 | 0 | <11 | <11 | 0 | 42 (0.4%) | 35 (0.3%) | 0.02 |
| Calendar year of index date, n (%) | | | | | | | | | |
| 2007 | 0 (0.0%) | 0 (0.0%) | 0 | 0 (0.0%) | 0 (0.0%) | 0 | 874 (7.3%) | 870 (7.3%) | 0 |
| 2008 | 0 (0.0%) | 0 (0.0%) | 0 | 0 (0.0%) | 0 (0.0%) | 0 | 784 (6.5%) | 775 (6.5%) | 0 |
| 2009 | 0 (0.0%) | 0 (0.0%) | 0 | 0 (0.0%) | 0 (0.0%) | 0 | 958 (8.0%) | 969 (8.1%) | 0 |
| 2010 | 0 (0.0%) | 0 (0.0%) | 0 | 62 (1.0%) | 64 (1.0%) | 0 | 972 (8.1%) | 944 (7.9%) | 0.01 |
| 2011 | 0 (0.0%) | 0 (0.0%) | 0 | 750 (11.8%) | 748 (11.7%) | 0 | 925 (7.7%) | 912 (7.6%) | 0 |
| 2012 | 15 (0.4%) | 17 (0.4%) | 0 | 574 (9.0%) | 566 (8.9%) | 0 | 1,073 (9.0%) | 1,036 (8.7%) | 0.01 |
| 2013 | 475 (11.2%) | 481 (11.4%) | -0.01 | 802 (12.6%) | 776 (12.2%) | 0.01 | 1,113 (9.3%) | 1,085 (9.1%) | 0.01 |

© 2022 Desai RJ et al. *JAMA Network Open.*

| | | | | | | | | | |
|---|---|---|---|---|---|---|---|---|---|
| 2014 | 807 (19.1%) | 831 (19.7%) | -0.02 | 898 (14.1%) | 920 (14.4%) | -0.01 | 1,006 (8.4%) | 1,026 (8.6%) | -0.01 |
| 2015 | 907 (21.5%) | 908 (21.5%) | 0 | 979 (15.4%) | 974 (15.3%) | 0 | 1,353 (11.3%) | 1,387 (11.6%) | -0.01 |
| 2016 | 859 (20.3%) | 840 (19.9%) | 0.01 | 1,035 (16.3%) | 1,047 (16.4%) | 0 | 1,399 (11.7%) | 1,401 (11.7%) | 0 |
| 2017 | 1,161 (27.5%) | 1,147 (27.2%) | 0.01 | 1,269 (19.9%) | 1,274 (20.0%) | 0 | 1,519 (12.7%) | 1,571 (13.1%) | -0.01 |
| Low income subsidy, n (%) | 1,396 (33.0%) | 1,383 (32.7%) | 0.01 | 966 (15.2%) | 974 (15.3%) | 0 | 2,080 (17.4%) | 2,103 (17.6%) | -0.01 |
| **Dementia risk factors, n (%)** | | | | | | | | | |
| Diabetes | 1,338 (31.7%) | 1,329 (31.5%) | 0 | 1,886 (29.6%) | 1,859 (29.2%) | 0.01 | 3,884 (32.4%) | 3,857 (32.2%) | 0 |
| Obesity | 808 (19.1%) | 812 (19.2%) | 0 | 1,102 (17.3%) | 1,084 (17.0%) | 0.01 | 1,720 (14.4%) | 1,685 (14.1%) | 0.01 |
| Hypertension | 3,269 (77.4%) | 3,276 (77.6%) | 0 | 4,843 (76.0%) | 4,833 (75.9%) | 0 | 9,357 (78.1%) | 9,358 (78.1%) | 0 |
| Coronary artery disease | 1,111 (26.3%) | 1,138 (26.9%) | -0.01 | 1,680 (26.4%) | 1,632 (25.6%) | 0.02 | 3,447 (28.8%) | 3,479 (29.0%) | 0 |
| Depression | 876 (20.7%) | 855 (20.2%) | 0.01 | 1,256 (19.7%) | 1,286 (20.2%) | -0.01 | 2,336 (19.5%) | 2,306 (19.3%) | 0.01 |
| Anxiety | 717 (17.0%) | 706 (16.7%) | 0.01 | 900 (14.1%) | 913 (14.3%) | -0.01 | 1,541 (12.9%) | 1,556 (13.0%) | 0 |
| Bipolar disorder | 44 (1.0%) | 53 (1.3%) | -0.03 | 72 (1.1%) | 75 (1.2%) | -0.01 | 98 (0.8%) | 107 (0.9%) | -0.01 |
| Schizophrenia | <11 | 12 (0.3%) | -0.02 | <11 | <11 | 0 | 15 (0.1%) | 16 (0.1%) | 0 |
| **Markers for healthy behavior, frailty, healthcare use** | | | | | | | | | |
| Smoking, n (%) | 1,116 (26.4%) | 1,130 (26.8%) | -0.01 | 1,491 (23.4%) | 1,478 (23.2%) | 0 | 2,188 (18.3%) | 2,206 (18.4%) | 0 |
| Mammography, n (%) | 1,415 (33.5%) | 1,390 (32.9%) | 0.01 | 2,185 (34.3%) | 2,204 (34.6%) | -0.01 | 3,875 (32.4%) | 3,887 (32.5%) | 0 |
| Colonoscopy, n (%) | 434 (10.3%) | 412 (9.8%) | 0.02 | 841 (13.2%) | 837 (13.1%) | 0 | 1,513 (12.6%) | 1,519 (12.7%) | 0 |
| Fecal occult blood test, n (%) | 367 (8.7%) | 369 (8.7%) | 0 | 597 (9.4%) | 615 (9.7%) | -0.01 | 1,171 (9.8%) | 1,177 (9.8%) | 0 |
| Influenza vaccination, n (%) | 2,791 (66.1%) | 2,772 (65.6%) | 0.01 | 4,412 (69.3%) | 4,454 (69.9%) | -0.01 | 8,092 (67.6%) | 8,120 (67.8%) | 0 |
| Pneumococcal vaccination, n (%) | 1,717 (40.6%) | 1,701 (40.3%) | 0.01 | 2,208 (34.7%) | 2,226 (35.0%) | -0.01 | 3,138 (26.2%) | 3,263 (27.2%) | -0.02 |
| Herpes zoster vaccination, n (%) | 25 (0.6%) | 26 (0.6%) | 0 | 22 (0.3%) | 31 (0.5%) | -0.03 | 77 (0.6%) | 77 (0.6%) | 0 |
| Bone mineral density test, n (%) | 1,060 (25.1%) | 1,092 (25.9%) | -0.02 | 1,853 (29.1%) | 1,860 (29.2%) | 0 | 3,863 (32.3%) | 3,882 (32.4%) | 0 |
| Number of distinct generic agents, mean (SD) | 15.81 (7.11) | 15.95 (7.40) | 0 | 14.48 (6.72) | 14.43 (6.63) | 0 | 14.52 (6.70) | 14.49 (6.62) | 0 |
| Number of emergency room visits, mean (SD) | 0.69 (1.37) | 0.70 (1.28) | 0 | 0.64 (1.21) | 0.63 (1.16) | 0 | 0.67 (1.25) | 0.67 (1.22) | 0 |
| Number of outpatient visits, mean (SD) | 18.77 (19.30) | 18.95 (14.06) | 0 | 20.22 (16.82) | 20.23 (16.90) | 0 | 20.83 (19.19) | 20.73 (17.76) | 0 |
| Number of hospitalizations, mean (SD) | 0.31 (0.84) | 0.31 (0.73) | 0 | 0.32 (0.72) | 0.31 (0.72) | 0 | 0.37 (0.80) | 0.37 (0.81) | 0 |
| Number of physician office visits, mean (SD) | 4.92 (9.75) | 5.00 (6.31) | 0 | 5.03 (8.93) | 5.05 (8.77) | 0 | 5.76 (10.01) | 5.69 (9.04) | 0 |

© 2022 Desai RJ et al. *JAMA Network Open.*

| | | | | | | | | | |
|---|---|---|---|---|---|---|---|---|---|
| Number of rheumatologist visits, mean (SD) | 4.44 (3.16) | 4.49 (3.14) | 0 | 5.49 (3.88) | 5.46 (4.02) | 0 | 4.96 (4.01) | 4.98 (3.69) | 0 |
| Number of C-reactive protein tests ordered, mean (SD) | 2.59 (2.30) | 2.61 (2.34) | 0 | 3.35 (2.92) | 3.35 (2.87) | 0 | 2.63 (2.57) | 2.66 (2.54) | 0 |
| Number of serum creatinine tests ordered, mean (SD) | 5.87 (3.42) | 5.95 (3.18) | 0 | 6.76 (3.65) | 6.72 (3.53) | 0 | 6.43 (3.81) | 6.43 (3.70) | 0 |
| Composite frailty score, mean (SD) | 0.17 (0.04) | 0.17 (0.04) | 0 | 0.17 (0.04) | 0.17 (0.04) | 0 | 0.17 (0.05) | 0.17 (0.04) | 0 |
| Osteoporosis, n (%) | 1,637 (38.8%) | 1,669 (39.5%) | -0.01 | 2,605 (40.9%) | 2,588 (40.6%) | 0.01 | 5,300 (44.3%) | 5,268 (44.0%) | 0.01 |
| Fractures, n (%) | 465 (11.0%) | 499 (11.8%) | -0.03 | 698 (11.0%) | 671 (10.5%) | 0.02 | 1,384 (11.6%) | 1,409 (11.8%) | -0.01 |
| Falls, n (%) | 207 (4.9%) | 205 (4.9%) | 0 | 304 (4.8%) | 291 (4.6%) | 0.01 | 569 (4.8%) | 555 (4.6%) | 0.01 |
| Use of supplemental oxygen, n (%) | 90 (2.1%) | 77 (1.8%) | 0.02 | 119 (1.9%) | 112 (1.8%) | 0.01 | 169 (1.4%) | 167 (1.4%) | 0 |
| Combined comorbidity score, mean (SD) | 1.60 (2.26) | 1.61 (2.25) | 0 | 1.58 (2.21) | 1.53 (2.19) | 0 | 1.72 (2.34) | 1.73 (2.31) | 0 |
| **Comedication use, n (%)** | | | | | | | | | |
| Lithium | <11 | <11 | 0 | <11 | <11 | 0 | 13 (0.1%) | <11 | 0 |
| Anti-epileptic mood stabilizers | 64 (1.5%) | 73 (1.7%) | -0.02 | 95 (1.5%) | 101 (1.6%) | -0.01 | 155 (1.3%) | 152 (1.3%) | 0 |
| Anti-epileptics (other than mood stabilizers) | 1,187 (28.1%) | 1,192 (28.2%) | 0 | 1,630 (25.6%) | 1,621 (25.5%) | 0 | 2,996 (25.0%) | 3,032 (25.3%) | -0.01 |
| Atypical antipsychotics | 93 (2.2%) | 93 (2.2%) | 0 | 105 (1.6%) | 101 (1.6%) | 0 | 192 (1.6%) | 185 (1.5%) | 0.01 |
| Benzodiazepines | 920 (21.8%) | 935 (22.1%) | -0.01 | 1,125 (17.7%) | 1,120 (17.6%) | 0 | 1,583 (13.2%) | 1,613 (13.5%) | -0.01 |
| Serotonin-norepinephrine reuptake inhibitors | 583 (13.8%) | 581 (13.8%) | 0 | 768 (12.1%) | 775 (12.2%) | 0 | 1,383 (11.5%) | 1,357 (11.3%) | 0.01 |
| Selective serotonin reuptake inhibitors | 835 (19.8%) | 818 (19.4%) | 0.01 | 1,351 (21.2%) | 1,384 (21.7%) | -0.01 | 2,444 (20.4%) | 2,494 (20.8%) | -0.01 |
| Tricyclic antidepressants | 252 (6.0%) | 256 (6.1%) | 0 | 460 (7.2%) | 453 (7.1%) | 0 | 884 (7.4%) | 890 (7.4%) | 0 |
| Typical antipsychotics | <11 | <11 | 0 | 12 (0.2%) | 14 (0.2%) | 0 | 28 (0.2%) | 33 (0.3%) | -0.02 |
| Anticoagulants | 449 (10.6%) | 455 (10.8%) | -0.01 | 718 (11.3%) | 709 (11.1%) | 0.01 | 1,365 (11.4%) | 1,397 (11.7%) | -0.01 |
| Antiplatelet agents | 346 (8.2%) | 351 (8.3%) | 0 | 495 (7.8%) | 500 (7.9%) | 0 | 1,118 (9.3%) | 1,110 (9.3%) | 0 |
| Angiotensin II receptor blockers | 1,079 (25.5%) | 1,078 (25.5%) | 0 | 1,522 (23.9%) | 1,556 (24.4%) | -0.01 | 2,882 (24.1%) | 2,840 (23.7%) | 0.01 |
| Angiotensin converting enzyme inhibitors | 1,115 (26.4%) | 1,133 (26.8%) | -0.01 | 1,737 (27.3%) | 1,740 (27.3%) | 0 | 3,417 (28.5%) | 3,436 (28.7%) | 0 |
| Beta blockers | 1,690 (40.0%) | 1,717 (40.6%) | -0.01 | 2,506 (39.3%) | 2,464 (38.7%) | 0.01 | 4,878 (40.7%) | 4,954 (41.4%) | -0.01 |
| Calcium channel blockers | 1,242 (29.4%) | 1,213 (28.7%) | 0.02 | 1,721 (27.0%) | 1,726 (27.1%) | 0 | 3,399 (28.4%) | 3,369 (28.1%) | 0.01 |
| Diuretics | 1,329 (31.5%) | 1,329 (31.5%) | 0 | 2,036 (32.0%) | 1,963 (30.8%) | 0.03 | 4,114 (34.4%) | 4,097 (34.2%) | 0 |
| Nitrates | 283 (6.7%) | 294 (7.0%) | -0.01 | 401 (6.3%) | 376 (5.9%) | 0.02 | 906 (7.6%) | 911 (7.6%) | 0 |
| Lipid lowering drugs | 2,125 (50.3%) | 2,191 (51.9%) | -0.03 | 3,159 (49.6%) | 3,136 (49.2%) | 0.01 | 5,860 (48.9%) | 5,865 (49.0%) | 0 |

© 2022 Desai RJ et al. *JAMA Network Open.*

| | | | | | | | | | |
|---|---|---|---|---|---|---|---|---|---|
| Non-insulin diabetes medications | 710 (16.8%) | 682 (16.1%) | 0.02 | 1,017 (16.0%) | 1,014 (15.9%) | 0 | 1,928 (16.1%) | 1,901 (15.9%) | 0.01 |
| Insulin | 290 (6.9%) | 290 (6.9%) | 0 | 402 (6.3%) | 388 (6.1%) | 0.01 | 785 (6.6%) | 759 (6.3%) | 0.01 |
| Antidepressants | 1,677 (39.7%) | 1,665 (39.4%) | 0.01 | 2,557 (40.1%) | 2,570 (40.4%) | -0.01 | 4,590 (38.3%) | 4,617 (38.6%) | -0.01 |
| **Rheumatoid arthritis related factors** | | | | | | | | | |
| Number of combined biologic DMARDs, n (%) | | | | | | | | | |
| 0 | 1,454 (34.4%) | 1,417 (33.5%) | 0.02 | 1,726 (27.1%) | 1,686 (26.5%) | 0.01 | 8,791 (73.4%) | 8,776 (73.3%) | 0 |
| 1 | 1,513 (35.8%) | 1,514 (35.8%) | 0 | 3,007 (47.2%) | 3,027 (47.5%) | -0.01 | 2,148 (17.9%) | 2,130 (17.8%) | 0 |
| 2 | 866 (20.5%) | 873 (20.7%) | 0 | 1,211 (19.0%) | 1,245 (19.5%) | -0.01 | 749 (6.3%) | 779 (6.5%) | -0.01 |
| 3 or more | 391 (9.3%) | 420 (9.9%) | -0.02 | 425 (6.7%) | 411 (6.5%) | 0.01 | 288 (2.4%) | 291 (2.4%) | 0 |
| Number of combined nonbiologic DMARDs, n (%) | | | | | | | | | |
| 0 | 396 (9.4%) | 416 (9.8%) | -0.01 | 642 (10.1%) | 622 (9.8%) | 0.01 | 1,079 (9.0%) | 1,055 (8.8%) | 0.01 |
| 1 | 1,542 (36.5%) | 1,494 (35.4%) | 0.02 | 2,657 (41.7%) | 2,678 (42.0%) | -0.01 | 4,723 (39.4%) | 4,624 (38.6%) | 0.02 |
| 2 | 1,312 (31.1%) | 1,311 (31.0%) | 0 | 1,875 (29.4%) | 1,885 (29.6%) | 0 | 3,679 (30.7%) | 3,800 (31.7%) | -0.02 |
| 3 or more | 974 (23.1%) | 1,003 (23.7%) | -0.01 | 1,195 (18.8%) | 1,184 (18.6%) | 0.01 | 2,495 (20.8%) | 2,497 (20.9%) | 0 |
| Use of other nonbiologic DMARDs, n (%) | 817 (19.3%) | 875 (20.7%) | -0.04 | 1,017 (16.0%) | 1,033 (16.2%) | -0.01 | 2,390 (20.0%) | 2,379 (19.9%) | 0 |
| Methotrexate, n (%) | 2,168 (51.3%) | 2,129 (50.4%) | 0.02 | 3,435 (53.9%) | 3,381 (53.1%) | 0.02 | 6,930 (57.9%) | 7,002 (58.5%) | -0.01 |
| Hydroxychloroquine, n (%) | 1,038 (24.6%) | 1,070 (25.3%) | -0.02 | 1,275 (20.0%) | 1,275 (20.0%) | 0 | 3,452 (28.8%) | 3,477 (29.0%) | 0 |
| Leflunomide, n (%) | 902 (21.4%) | 888 (21.0%) | 0.01 | 1,151 (18.1%) | 1,193 (18.7%) | -0.02 | 2,471 (20.6%) | 2,492 (20.8%) | 0 |
| Nonsteroidal anti-inflammatory drugs/Coxibs, n (%) | 1,474 (34.9%) | 1,472 (34.8%) | 0 | 2,050 (32.2%) | 2,022 (31.7%) | 0.01 | 4,127 (34.5%) | 4,119 (34.4%) | 0 |
| Opioids, n (%) | 2,870 (67.9%) | 2,877 (68.1%) | 0 | 4,544 (71.3%) | 4,502 (70.7%) | 0.01 | 8,799 (73.5%) | 8,742 (73.0%) | 0.01 |
| Glucocorticoids use/dose, n (%) | 3,127 (74.0%) | 3,099 (73.4%) | 0.01 | 4,913 (77.1%) | 4,877 (76.6%) | 0.01 | 8,880 (74.1%) | 8,943 (74.7%) | -0.01 |
| **Comorbid conditions, n (%)** | | | | | | | | | |
| Atrial fibrillation | 451 (10.7%) | 435 (10.3%) | 0.01 | 741 (11.6%) | 720 (11.3%) | 0.01 | 1,514 (12.6%) | 1,528 (12.8%) | -0.01 |
| Heart failure | 522 (12.4%) | 526 (12.5%) | 0 | 748 (11.7%) | 697 (10.9%) | 0.03 | 1,748 (14.6%) | 1,696 (14.2%) | 0.01 |
| Stroke or transient ischemic attack | 343 (8.1%) | 343 (8.1%) | 0 | 600 (9.4%) | 599 (9.4%) | 0 | 1,098 (9.2%) | 1,086 (9.1%) | 0 |
| Peripheral vascular disease | 542 (12.8%) | 537 (12.7%) | 0 | 715 (11.2%) | 696 (10.9%) | 0.01 | 1,589 (13.3%) | 1,578 (13.2%) | 0 |
| Hyperlipidemia | 2,824 (66.9%) | 2,834 (67.1%) | 0 | 4,390 (68.9%) | 4,344 (68.2%) | 0.02 | 8,165 (68.2%) | 8,199 (68.5%) | -0.01 |
| Renal dysfunction | 721 (17.1%) | 723 (17.1%) | 0 | 1,044 (16.4%) | 1,034 (16.2%) | 0.01 | 1,972 (16.5%) | 1,986 (16.6%) | 0 |

© 2022 Desai RJ et al. *JAMA Network Open.*

| | | | | | | | | | |
|---|---|---|---|---|---|---|---|---|---|
| Chronic liver disease | 361 (8.5%) | 362 (8.6%) | 0 | 571 (9.0%) | 561 (8.8%) | 0.01 | 1,224 (10.2%) | 1,238 (10.3%) | 0 |
| Asthma | 940 (22.3%) | 939 (22.2%) | 0 | 1,253 (19.7%) | 1,252 (19.7%) | 0 | 1,985 (16.6%) | 1,987 (16.6%) | 0 |
| Ischemic heart disease | 1,080 (25.6%) | 1,103 (26.1%) | -0.01 | 1,626 (25.5%) | 1,576 (24.7%) | 0.02 | 3,348 (28.0%) | 3,388 (28.3%) | -0.01 |
| Chronic obstructive pulmonary disease | 1,135 (26.9%) | 1,111 (26.3%) | 0.01 | 1,733 (27.2%) | 1,752 (27.5%) | -0.01 | 2,986 (24.9%) | 3,031 (25.3%) | -0.01 |
| Malignancy | 901 (21.3%) | 904 (21.4%) | 0 | 1,674 (26.3%) | 1,698 (26.7%) | -0.01 | 3,093 (25.8%) | 3,023 (25.2%) | 0.01 |
| Drug or alcohol abuse or dependence | 490 (11.6%) | 480 (11.4%) | 0.01 | 606 (9.5%) | 627 (9.8%) | -0.01 | 916 (7.6%) | 924 (7.7%) | 0 |
| Venous thromboembolism | 243 (5.8%) | 249 (5.9%) | 0 | 381 (6.0%) | 382 (6.0%) | 0 | 796 (6.6%) | 762 (6.4%) | 0.01 |

* Numbers <11 are suppressed according to Data Use Agreement with the Centers for Medicare and Medicare Services

© 2022 Desai RJ et al. *JAMA Network Open.*

**eTable 4.** Results for Alzheimer Disease in Patients Treated With Targeted Disease Modifying Antirheumatic Drugs

| Analysis scheme* | Exposure | N of patients | N outcomes† | N person years | Avg follow-up (years) | Incidence rate (95% CI)/1,000 py | Hazard Ratio (95%CI) |
|---|---|---|---|---|---|---|---|
| **Tofacitinib vs abatacept** | | | | | | | |
| Analysis 1 | Tofacitinib | 4,224 | <11 | 3,471 | 0.8 | 2.02 (0.81-4.16) | 1.06 (0.38, 2.93) |
| | Abatacept | 4,224 | <11 | 3,994 | 0.9 | 2.00 (0.86-3.95) | |
| Analysis 2 | Tofacitinib | 2,164 | 11 | 3,393 | 1.6 | 3.24 (1.62-5.80) | 0.97 (0.43, 2.19) |
| | Abatacept | 2,164 | 12 | 3,521 | 1.6 | 3.41 (1.76-5.95) | |
| Analysis 3 | Tofacitinib | 2,053 | <11 | 2,032 | 1.0 | 3.44 (1.38-7.10) | 1.80 (0.53-6.16) |
| | Abatacept | 2,053 | <11 | 2,063 | 1.0 | 1.94 (0.53-4.96) | |
| Analysis 4 | Tofacitinib | 4,224 | <11 | 3,472 | 0.8 | 1.73 (0.63-3.76) | 1.89 (0.53, 6.72) |
| | Abatacept | 4,224 | <11 | 3,996 | 0.9 | 1.00 (0.27-2.56) | |
| **Tocilizumab vs abatacept** | | | | | | | |
| Analysis 1 | Tocilizumab | 6,369 | <11 | 7,031 | 1.1 | 1.42 (0.68-2.62) | 0.65 (0.30, 1.41) |
| | Abatacept | 6,369 | 17 | 7,492 | 1.2 | 2.27 (1.32-3.63) | |
| Analysis 2 | Tocilizumab | 3,949 | 29 | 7,953 | 2.0 | 3.65 (2.44-5.24) | 1.45 (0.82, 2.57) |
| | Abatacept | 3,949 | 20 | 7,960 | 2.0 | 2.51 (1.53-3.88) | |
| Analysis 3 | Tocilizumab | 3,494 | <11 | 4,568 | 1.3 | 2.19 (1.05-4.02) | 1.03 (0.43-2.48) |
| | Abatacept | 3,494 | <11 | 4,690 | 1.3 | 2.13 (1.02-3.92) | |
| Analysis 4 | Tocilizumab | 6,369 | <11 | 7,034 | 1.1 | 1.00 (0.40-2.05) | 0.86 (0.32, 2.32) |
| | Abatacept | 6,369 | <11 | 7,500 | 1.2 | 1.20 (0.55-2.28) | |
| **TNFi vs abatacept** | | | | | | | |
| Analysis 1 | TNFi | 11,976 | 27 | 15,427 | 1.3 | 1.75 (1.15-2.55) | 0.79 (0.48, 1.31) |
| | Abatacept | 11,976 | 36 | 16,243 | 1.3 | 2.22 (1.55-3.07) | |
| Analysis 2 | TNFi | 7,940 | 71 | 17,591 | 2.2 | 4.04 (3.15-5.09) | 1.13 (0.80, 1.59) |
| | Abatacept | 7,940 | 63 | 17,632 | 2.2 | 3.57 (2.75-4.57) | |
| Analysis 3 | TNFi | 7127 | 21 | 11,304 | 1.6 | 1.86 (1.14-2.84) | 0.78 (0.44-1.37) |
| | Abatacept | 7127 | 28 | 11,592 | 1.6 | 2.41 (1.60-3.49) | |
| Analysis 4 | TNFi | 11,976 | 20 | 15,432 | 1.3 | 1.30 (0.79-2.00) | 1.12 (0.60, 2.09) |
| | Abatacept | 11,976 | 19 | 16,263 | 1.4 | 1.17 (0.70-1.82) | |

*Analysis 1: ‘As-treated’ follow-up approach; Analysis 2: ‘As-started’ follow-up approach incorporating a 6-month induction period; Analysis 3: Incorporating a 6-month ‘symptom to diagnosis’ period’ and Analysis 4: Alternate outcome definition (See Methods for additional description of analytic approach).
† Numbers <11 are suppressed according to Data Use Agreement with the Centers for Medicare and Medicare Services

© 2022 Desai RJ et al. *JAMA Network Open.*
